# Age‐Related Lamin B1 Deficiency Activates SLC7A11‐Dependent Disulfidptosis to Impair Osteogenesis via Intercepting Mitochondrial Fission

**DOI:** 10.1002/advs.78203

**Published:** 2026-10-08

**Authors:** Wenhui Yu, Qibo Li, Weihao Zhang, Guan Zheng, Junhua Chen, Yipeng Zeng, Zipeng Xiao, Zibin Chen, Ziqian Liu, Yangfeng Lin, Jiajie Lin, Zepeng Su, Zhongyu Xie

**Affiliations:** ^1^ Department of Orthopedics The Eighth Affiliated Hospital of Sun Yat‐Sen University Shenzhen P. R. China; ^2^ Guangdong Provincial Clinical Research Center for Orthopedic Diseases Shenzhen P. R. China; ^3^ Center For Biotherapy the Eighth Affiliated Hospital of Sun Yat‐Sen University Shenzhen P. R. China

**Keywords:** Disulfidptosis, Lamin B1, Mitochondria, Osteogenesis, Senile osteoporosis, SLC7A11

## Abstract

Senile osteoporosis (SOP) is an aging‐related disease characterized by decreased bone mass and susceptibility to fracture. The pathological mechanism of SOP, which involves impaired osteogenesis of bone marrow‐derived mesenchymal stem cells (MSCs), warrants further investigation to facilitate the development of novel therapeutic agents. In this study, we demonstrated that age‐related lamin B1 (LMNB1) deficiency in SOP‐MSCs led to lamina‐associated domain (LAD) detachment, causing super‐enhancer (SE) formation to effectively drive SLC7A11 expression. Elevated SLC7A11 expression activated disulfidptosis in SOP‐MSCs, thereby resulting in disulfide bond formation in actin and subsequent cytoskeleton collapse. The disulfide‐crosslinked actin‐induced cytoskeleton disorganization further disrupted their binding with DRP1, inducing mitophagy and oxidative phosphorylation dysfunction by intercepting mitochondrial fission. The resulting mitochondrial disorders impaired the osteogenesis of SOP‐MSCs, aggravating age‐related bone loss in SOP. Moreover, we identified the FDA‐approved drug naldemedine as a potent inhibitor of SLC7A11‐dependent disulfidptosis through virtual molecular screening and confirmed its therapeutic potential for SOP. Our findings explore the connection among age‐related LMNB1 deficiency, SLC7A11‐dependent disulfidptosis and mitochondrial fission disruption in SOP‐MSCs and reveal their roles in the molecular mechanism of impaired osteogenesis in SOP. These data contribute to a better understanding of SOP pathogenesis and provide insight into potential clinical treatments.

## Graphical abstract

This study reveals that age‐related LMNB1 deficiency in mesenchymal stem cells epigenetically upregulates SLC7A11, triggering actin disulfidptosis and mitochondrial dysfunction. This cascade impairs bone formation in senile osteoporosis. Furthermore, researchers identified the FDA‐approved drug naldemedine as a targeted SLC7A11 inhibitor to reverse bone loss. These findings highlight a novel pathological mechanism and promising therapeutic strategy.

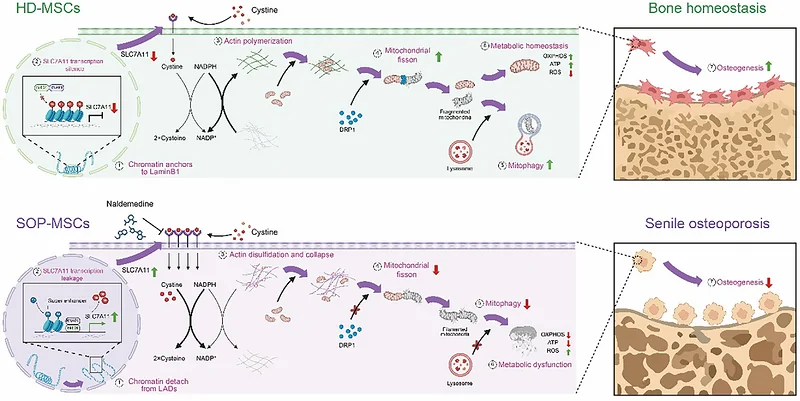

## 1 Introduction

Senile osteoporosis (SOP) is an age‐related skeletal disease characterized by reduced bone mass and compromised bone microarchitecture, a condition largely driven by the balance shifting from bone formation toward bone resorption with advancing age [1]. To mitigate the high risk of fractures, osteoblast‐stimulating anabolic agents and osteoclast‐inhibiting antiresorptive drugs are recommended as therapies for SOP patients [2]. However, the clinical choice of osteoanabolic agents is still limited, as current FDA‐approved therapies such as teriparatide and romosozumab require frequent subcutaneous injections and entail high costs, frequently leading to poor long‐term patient compliance. Therefore, revealing the mechanism of impaired osteogenesis in SOP, which is driven by the decreased osteogenic differentiation capacity of bone marrow‐derived mesenchymal stem cells (MSCs) [3, 4, 5], can provide novel insight into the development of osteoanabolic agents.

Age‐related diseases, including senile osteoporosis, are frequently associated with aberrant epigenetic landscape alterations mediated by a series of cellular senescence markers, leading to increased chromatin accessibility and disordered transcriptional activation [6, 7]. Deficiency in lamin B1 (LMNB1), a key component of the nuclear lamina, is a recognized hallmark of aging [8]. LMNB1 normally interacts with chromatin to form lamina‐associated domains (LADs) and maintain heterochromatin in a condensed, transcriptionally repressive state [9], while LMNB1 deficiency under senile conditions results in LAD detachment and the subsequent transcriptional leakage of normally silenced genes, thus disrupting cellular functions in age‐related diseases [10, 11, 12]. As chromatin organization and epigenetic programming greatly affect the functions of MSCs [13, 14], LMNB1 is highly likely to play a role in the impaired osteogenesis of MSCs in SOP.

Solute carrier family 7 member 11 (SLC7A11), a cystine/glutamate antiporter, imports extracellular cystine into the cytoplasm to synthesize glutathione and consumes substantial amounts of nicotinamide adenine dinucleotide phosphate (NADPH) [15]. Under conditions of insufficient NADPH supply, cystine aberrantly accumulates in the cell, especially those with enhanced SLC7A11 expression, and then results in disulfide bond formation in actin proteins, cytoskeleton collapse and ultimately disulfidptosis [16, 17]. Alterations in NADPH homeostasis can be found in virtually all age‐related diseases, including osteoporosis [18, 19], but whether SLC7A11‐mediated disulfidptosis is involved in the impaired osteogenesis of SOP remains entirely unexplored.

Mitochondrial fission physically segregates the damaged segments from the healthy network and renders them suitable for mitophagy, a pivotal mitochondrial quality control mechanism to maintain cellular bioenergetic homeostasis by orchestrating the lysosomal degradation of dysfunctional mitochondria [20]. During mitochondrial fission, the F‐actin scaffold is required for the recruitment and stabilization of DRP1 to form a contractile restriction ring at mitochondrial fission sites [21]. However, the disruption of this process in age‐related diseases disturbs fission‐mitophagy flux and subsequent mitochondrial bioenergetic homeostasis, therefore affecting the functions of various stem cells [22, 23, 24]. Mitochondrial bioenergetic homeostasis is essential for the osteogenic differentiation of MSCs [25], but the detailed role and intrinsic mechanism underlying mitochondrial fission in SOP‐MSCs warrant further investigation.

In this study, we demonstrated that LMNB1 deficiency led to chromatin detachment from LADs and epigenetic reprogramming, therefore driving super‐enhancer (SE)‐related SLC7A11 upregulation in SOP‐MSCs. Moreover, we determined that SLC7A11‐dependent disulfidptosis in SOP‐MSCs triggered disulfide bond formation in actin and cytoskeleton collapse and then impaired the osteogenic differentiation capacity of SOP‐MSCs by intercepting DRP1‐mediated mitochondrial fission. Significantly, by utilizing *LMNB1^fl/fl^ Prx1‐cre* (hereafter *LMNB1^△Prx1^*), *SLC7A11^fl/fl^ Prx1‐cre* (hereafter *SLC7A11^△Prx1^*) and LMNB1 and SLC7A11 double conditional knockout (dCKO) mice, we further substantiated the therapeutic potential of this molecular mechanism to reverse age‐related bone loss in SOP. Using molecular docking, we identified that the FDA‐approved drug naldemedine could effectively serve as an anabolic therapy for SOP by targeting SLC7A11‐dependent disulfidpotosis. These findings reveal a novel pathogenesis concerning age‐related LMNB1 deficiency, SLC7A11‐dependent disulfidptosis and mitochondrial fission disruption and provide a promising therapeutic strategy for SOP.

## 2 Methods and Materials

### 2.1 Study Approval

This study complies with all relevant ethical regulations of the Ethics Committee of the Eighth Affiliated Hospital of Sun Yat‐Sen University (No. ZDBY2024r038) and the Institutional Animal Care and Use Committee of Sun Yat‐Sen University (No. SYSU 2024‐065‐01). All procedures were performed in strict adherence to the rules and guidelines for the ethical use of animals in research. A total of 23 old subjects with SOP and 17 young healthy donors (HD) without OP (Table S2) were recruited. SOP was diagnosed according to a BMD T score of less than ‐2.5 at the lumbar spine by dual‐energy X‐ray absorptiometry. After signed informed consent, the bone tissues in the vertebra were acquired during the surgery, and bone marrow punctures were performed.

### 2.2 MSC Isolation and Culture

To isolate MSCs from human subjects, bone marrow was extracted from HD and SOP patients through bone puncture as previously described [26]. The bone marrow was subjected to density gradient centrifugation at 12,00 rpm for 30 min to isolate MSCs. The isolated MSCs were resuspended in Dulbecco's modified Eagle's medium (DMEM, with 1000 mg/L glucose, Gibco) containing 10% fetal bovine serum (FBS, Hangzhou Sijiqing Biological Engineering Material Company, Limited) and cultured in a 25 cm^2^ cell culture flask at 37°C in a 5% CO2 atmosphere. MSCs in the second passages were used for the subsequent experiments.

To isolate MSCs from murine subjects, femurs were collected, cut and crushed into pieces. After filtration through a 40‐µm cell strainer (BD, 352340), the filtrate was resuspended using the MesenCult Expansion Kit (Stemcell, 05513), seeded in 25 cm^2^ flasks and cultured at 37°C in a 5% CO_2_ atmosphere. The MSCs adhered to the flask, and nonadherent cells were removed after five days. MSCs in the third passage were used for subsequent experiments.

MSC osteogenic differentiation was induced using osteogenic medium consisting of DMEM (with 1000 mg/L glucose) supplemented with 10% FBS, 100 IU/mL penicillin, 100 IU/mL streptomycin, 0.1 µM dexamethasone, 10 mM β‐glycerol phosphate, and 50 µM ascorbic acid.

### 2.3 Construction and Reagents

sh‐LMNB1, sh‐SLC7A11, ov‐LMNB1, ov‐SLC7A11 lentiviruses and their controls were constructed by OBiO Technology, AAV9‐Flex‐shSLC7A11, lentivirus overexpressing MT‐mKeima‐Red‐Mito and enCRISPRi (dCas9‐KRAB + sgRNA‐MS2‐SV40‐MCP‐LSD1) were constructed by Genechem (Shanghai, China). See Table S2 for detailed information.

### 2.4 Animal Models

Mice were maintained under standard housing conditions (22 ± 2°C, 55% ± 10% humidity, 12 h light/dark cycle) with free access to food and water and were acclimatized for one week before experimentation. C57BL/6 mice were purchased from the Laboratory Animal Center of Sun Yat‐Sen University. *LMNB1^△Prx1^* and *SLC7A11^△Prx1^* CKO mice were constructed and bred by Shanghai Model Organisms Center, Inc. The following primers were used for genotyping: *LMNB1*: 5’ arm primers, forward, TCGGGGAGTTTCAGATCACC, and 5’ arm primers reverse, TGACTGTGGGACAAAGACCC. WT: one band with 294 bp; Heterozygous: two bands with 353 and 294 bp; Homozygous: one band with 353 bp.

*SLC7A11*: 5’ arm primers, forward, GGGTTGGTCTCTGGTGATCC, and 5’ arm primer reverse, TCAAAGCCAAGTGAGTGGTCT. WT: one band with 299 bp; Heterozygous: two bands with 358 and 299 bp; Homozygous: one band with 358 bp.

*Prx1*‐cre: forward, GGGTCTGTAAAACGTCAGGC, and reverse, GTGAAACAGCATTGCTGTCACTT, Tg: one band with 500 bp.

To construct dCKO mice with conditional knockout of LMNB1 and SLC7A11 in MSCs, AAV9‐Flex‐shSLC7A11 was diluted in sterile phosphate‐buffered saline (PBS) to a final volume of 200 µL and injected systemically via the tail vein of *LMNB1^△Prx1^* mice. *LMNB1^△Prx1^* mice were anesthetized with 2%–3% isoflurane and restrained in a dedicated apparatus, and the lateral tail vein was dilated by 70% ethanol sterilization. The viral suspension was slowly administered at the distal third of the tail. Digital compression was applied for 60 s post‐injection to achieve hemostasis prior to recovery.

### 2.5 Calvarial Defect Mice

Calvarial bone defects were created in the parietal bones of eight‐week‐old mice using an electric bone drill. Following anesthetization and disinfection, the skin was incised with a scalpel, and the subcutaneous tissue was meticulously peeled back to reveal the parietal bones. An electric bone drill equipped with a sterilized 2.5‐mm drill bit was then employed to penetrate the skull. After successfully breaching the skull, bone fragments were carefully extracted, and the incision was sutured with a fine thread. Eight weeks later, the entire skulls were harvested for micro‐CT analysis.

### 2.6 Micro‐CT

Micro‐CT analysis was conducted to evaluate bone structures using the Inveon MM system (Siemens). Images were acquired at 360 rotational steps, with a pixel size of 8.82 µm, a voltage of 80 kV, a current of 500 µA, and an exposure time of 1500 ms. Bone parameters, including bone volume/total volume (BV/TV), trabecular thickness (Tb. Th), trabecular number (Tb. N), cortical bone thickness (Ct. Th), and trabecular spacing (Tb. Sp), were calculated using the Inveon Research Workplace (Siemens) following established guidelines. Both 2D and 3D image slices of the bone structure were reconstructed.

### 2.7 Histology Staining

Mouse tibial specimens or HA/TCP grafts were fixed in 4% paraformaldehyde (24 hr, 4°C), decalcified in 20% EDTA, and paraffin‐embedded. Sectioned samples were subjected to H&E staining (BOSTER, AR1180) and Masson's trichrome (Solarbio, G1340‐100) according to standardized protocols. Immunohistochemistry was performed using an anti‐OSX antibody (Abcam. ab209484) or anti‐collagen I (Abcam, ab34710).

### 2.8 Immunofluorescence

Bone tissues were collected for immunofluorescence analysis. For antigen retrieval, bone tissue sections were deparaffinized and rehydrated, immersed in 10 mM citrate buffer (pH 6.0) and microwaved for 15 min. Bone sections were then permeabilized with 0.5% Triton X‐100 for 20 min and blocked for 1 h with 10% goat serum in PBS. After incubation with anti‐LMNB1 (Abcam, ab133741), anti‐CD90 (Abcam, ab92522), anti‐LEPR (Bioss, bs‐20498R), and anti‐SLC7A11 (Abcam, ab307601) primary antibodies overnight at 4°C, the samples were incubated for 1 h at room temperature with the following respective secondary antibodies: anti‐mouse Alexa 488 (Abcam, 150113), anti‐mouse Alexa 555 (Abcam, 150118), anti‐rabbit Alexa 488 (Abcam, ab150077) and anti‐rabbit Alexa 555 (Abcam, ab150078).

### 2.9 Super‐Resolution Fluorescence Imaging

Super‐resolution imaging was performed using a Nikon N‐STORM super‐resolution microscope (Nikon Instruments, Japan). MSCs were prepared for imaging as follows: fluorescent labeling of MSCs was achieved by FITC phalloidin (Solarbio, CA1620) at 100 nM and MitoTracker Deepred (Beyotime, C1034) at 200 nM. For DRP1 labeling, MSCs were fixed in 4% paraformaldehyde (PFA) for 15 min, permeabilized with 0.5% Triton X‐100 for 20 min and blocked for 1 h with 10% fetal bovine serum in PBS. After incubation with anti‐DRP1 (Abcam, ab184247), the samples were incubated for 1 h at room temperature with anti‐rabbit Alexa 555 (Abcam, ab150078).

Imaging was carried out using a 100× oil‐immersion objective (NA 1.49). Sequential acquisition for multiple channels was conducted using the appropriate laser lines. The acquired image stacks were then processed and analyzed using the manufacturer's software (NIS‐Elements) to reconstruct the final super‐resolution image.

### 2.10 Alizarin Red S (ARS) and Alkaline Phosphatase (ALP) Staining

Prior to staining, MSCs were washed twice with PBS and subsequently fixed in 4% paraformaldehyde (PFA) for 30 min. For ARS staining, calcium deposits in the MSCs were visualized by incubation in 1% ARS solution (pH 4.2) (Solarbio, G8550) for 15 min at room temperature. Next, the cells were rinsed twice with PBS to eliminate any nonspecific staining, and images were captured using a microscope. To remove excess ARS staining, the cells were subjected to destaining with 10% cetylpyridinium chloride monohydrate (Sigma–Aldrich, 8400080100) for 1 h at room temperature. The resulting extraction mixture (200 µL) was transferred to a 96‐well plate, and the mineral content was quantified at 562 nm using a microplate reader.

For ALP staining, MSCs were treated with a 5‐bromo‐4‐chloro‐3‐indolyl phosphate (BCIP)/nitro blue tetrazolium (NBT) alkaline phosphatase kit (Beyotime Institute of Biotechnology, C3206) following the manufacturer's instructions. Stained cells were photographed for documentation. For the ALP activity assay, MSCs were collected and lysed in ice‐cold RIPA buffer (Sigma–Aldrich, R0278). After centrifugation at 12,000 rpm and 4°C for 30 min, the protein concentration in the supernatant was measured using a bicinchoninic acid (BCA) assay kit (Solarbio, PC0020). ALP activity was assessed according to the manufacturer's instructions using ALP activity kits (Nanjing Jiancheng Biotech, Nanjing, China, A059‐2). The supernatants were incubated with reaction buffer at 37°C, and a stop solution was added after 15 min to halt the reaction. ALP activity was measured at 405 nm and normalized to the total protein concentration. The results are expressed as units per gram of protein per 15 min (U/g_pro/15 min).

### 2.11 Protein Extraction and Western Blotting

Whole‐cell proteins were extracted by lysing cells in ice‐cold RIPA buffer (Sigma, R0278) followed by centrifugation (12,000 rpm, 30 min, 4°C). Subcellular fractionation used NE‐PER reagents (Invitrogen, 78833) for nuclear/cytoplasmic proteins and mitochondrial isolation kits (Beyotime, C3601) for mitochondrial proteins. Protein samples were mixed with: non‐reducing buffer (Beyotime, P0016P) for actin disulfide analysis or standard loading buffer (Beyotime, P0015) for other targets. After SDS‒PAGE (10%) and PVDF transfer (Millipore IPVH00010), membranes were blocked in TBST/5% BSA, incubated overnight with primary antibodies (4°C), and then with HRP‐conjugated secondary antibodies for 1 h at room temperature (Cell Signaling Technology, 7076/7074). Signals were detected by chemiluminescence (Millipore, WBKLS0500). The following primary antibodies were used: anti‐RUNX2 (Cell Signaling Technology, 12556S), anti‐OSX (Abcam, ab209484), anti‐Collagen I (Abcam, ab34710), anti‐GAPDH (Cell Signaling Technology, 5174S), anti‐Lamin B1 (Abcam, ab133741), anti‐SLC7A11 (Abcam, ab307601), anti‐H3K27ac (Abcam, ab177178), anti‐BRD4 (Abcam, ab128874), anti‐POLII (Abcam, ab26721), anti‐H3K9me2 (Abcam, ab32521), anti‐H3K9me3 (Abcam, ab8898), anti‐H3 (Cell Signaling Technology, 4499T), anti‐ACTIN (Abcam, ab227387), anti‐DRP1 (Abcam, ab184247), anti‐FIS1 (ABclonal, A19666), anti‐MFN1 (ABclonal, A21293), anti‐MFN2 (ABclonal, A19678), and anti‐HSP60 (Abcam, ab45134).

### 2.12 RNA‐seq Library Preparation and Sequencing

Total RNA was isolated with RNAiso Plus (TaKaRa, 9109). PolyA^+^ mRNA was enriched using oligo(dT) magnetic beads and fragmented. First‐strand cDNA was synthesized with random hexamers, followed by second‐strand generation. End repair/A‐tailing and adapter ligation were performed prior to PCR amplification (AMPure XP‐purified). Library quality was verified on an Agilent 2100 bioanalyzer. Circularized ssDNA libraries were amplified by phi29 to generate DNA nanoballs (DNBs), which were sequenced (50‐bp single‐end) on a BGISeq500 platform (BGI‐Shenzhen).

### 2.13 RNA‐seq Data Analysis

The sequencing data were processed with SOAPnuke (v1.5.2) to filter out reads containing sequencing adapters, those with a low‐quality base rate (defined as a base quality less than or equal to 5) exceeding 20%, or those with an unknown base (‘N’) rate greater than 5%. Clean reads were then obtained and stored in FASTQ format. These clean reads were mapped to the hg38 reference genome for coverage calculation and quality control using HISAT2 (v2.0.4). Alignment of the clean reads to the hg38 reference genome was performed with Bowtie2 (v2.5.1), and gene expression levels were calculated using RSEM software (v1.2.12). A heatmap illustrating gene expression across different samples was created using the pheatmap package (v1.0.8) (available at https://CRAN.R‐project.org/package = pheatmap). Differential expression analysis was conducted with DESeq2 (v1.4.5) using a cutoff Q value of ≤ 0.05.

Kyoto Encyclopedia of Genes and Genomes (KEGG, https://www.kegg.jp/) and Gene Ontology (GO, http://www.geneontology.org/) enrichment analyses for the annotated differentially expressed genes were performed via the R package clusterProfiler (v3.11). The significance of terms and pathways was corrected according to the Q value using the Bonferroni method, with a strict threshold of Q value ≤ 0.05. Additionally, gene set enrichment analysis (GSEA) was carried out using OmicStudio tools, following the provided instructions at https://www.omicstudio.cn/tool.

### 2.14 High‐content Fluorescence Imaging

MSCs were seeded on 96‐well CellCarrier Ultra imaging plates (PerkinElmer, 6055300) before imaging. Images were acquired using the Operetta CLS High‐Content Analysis System (PerkinElmer) with 40× Air/0.6 NA. Fluorescent labeling of MSCs was achieved by TRITC phalloidin (100 nM, CA1610), MitoTracker Green (Beyotime, C1048) at 200 nM and CellROX (Invitrogen, C10422) at 5 µM for 1 h at 37°C and 5% CO2.

### 2.15 Cytoskeleton Network Analysis

Nuclei and cellular segmentation were defined using the “Find Nuclei” building block with the DAPI channel and the “Find Cytoplasm” building block with the TRITC Phalloidin channel. The cytoskeleton network was analyzed using SER texture properties (Ridge). F‐actin anisotropy was analyzed using the plugin FibrilTool of ImageJ.

### 2.16 Mitochondrial Morphology Analysis

Nuclei and cellular segmentation were defined using the “Find Nuclei” building block with the DAPI channel and the “Find Cytoplasm” building block with the MitoTracker Green channel. A supervised machine learning algorithm was used to identify the best properties able to segregate the three populations: “Fragmented”, “Intermediate” and “Tubulated” networks. Automatic single‐cell classification of nontraining samples was carried out by the supervised machine learning module. The mitochondrial network was analyzed using the MiNA plugin of ImageJ.

#### 2.16.1 Transmission Electron Microscopy (TEM)

MSCs were fixed in TEM fixative (4°C), pelleted (1,500 rpm, 5 min), and washed in 0.1 M PB (pH 7.4). Samples were embedded in 1% agarose, post‐fixed with 1% OsO4 (0.1 m PB, 2 h RT), and then dehydrated via an ethanol gradient (30%–100%) and acetone. Stepwise resin infiltration used acetone and EMBed 812 mixtures (1:1→1:2→pure) at 37°C. After polymerization (60°C, 48 h), ultrathin sections (60–80 nm) on copper grids were dual‐stained with 2% uranyl acetate (10 min dark) and lead citrate (10 min CO2‐free). The grids were observed under a HITACHI TEM (HT7800) for imaging.

### 2.17 ATAC‐seq and Data Processing

ATAC‐seq libraries were prepared using the ATAC‐Seq Library Prep Kit for Illumina (Novoprotein Scientific Inc., 12208ES). Briefly, cells were harvested and counted. Approximately 50,000 cells were pelleted and washed with cold wash buffer. The cell pellet was resuspended in 50 µL of ice‐cold lysis buffer (48.5 µL of suspension buffer, 0.5 µL of 10% Tween‐20, 0.5 µL of 10% NP‐40, and 0.5 µL of 1% digitonin) and incubated on ice for 5 min to isolate nuclei. After centrifugation, the nuclear pellet was subjected to tagmentation by resuspending in 50 µL of Transposition Mix (25 µL Reaction Buffer, 2 µL Transposome, 16.5 µL Wash Buffer, 0.5 µL 1% Digitonin, 0.5 µL 10% Tween‐20, and nuclease‐free water). The tagmentation reaction was incubated at 37°C for 30 min in a thermal cycler and then terminated with 5 µL of Terminate Solution. The tagmented DNA was purified using DNA extract beads and amplified by PCR using dual‐indexed primers. The final libraries were eluted in nuclease‐free water and stored at −20°C prior to sequencing.

The libraries were sequenced using an Illumina NovaSeq 6000 platform at Novogene Science and Technology Co., Ltd. (Beijing, China), generating 150 bp paired‐end reads. Initially, raw reads were filtered and trimmed to eliminate sequencing adapters and low‐quality reads using TrimGalore (v0.6.6) with the parameters ‐q 20 –phred33 –stringency 3. The clean reads were then mapped to the human genome reference hg38 with Bowtie2 (v2.5.1) using the default parameter. Peak calling was conducted with MACS2 (v2.1.1) using the parameters ‐p 1e‐5 ‐g hs –shift 75 –extsize 150 –nomodel. Heatmaps were created using the computeMatrix and plotHeatmap commands in deepTools (v2.3.6.0).

#### 2.17.1 Cleavage Under Targets and Tagmentation (CUT&Tag) Assay

The CUT&Tag assay was conducted using the NovoNGS CUT&Tag 4.0 High‐Sensitivity Kit (for Illumina) (Novoprotein Scientific Inc., N259‐YH01‐01A) as previously described with modifications [27]. In summary, cells were harvested by trypsinization and enriched using ConA magnetic beads. A total of 50,000 cells were resuspended and washed twice with 100 µL of Dig‐wash Buffer (20 mM HEPES, pH 7.5; 150 mM NaCl; 0.5 mM spermidine; 1× protease inhibitor cocktail; 0.05% digitonin). The samples were then incubated with a primary antibody (1:100 dilution, 4°C for 18 h) followed by a secondary antibody (1:200 dilution, at 25°C for 1 h). After incubation, the beads were washed three times with Dig‐Hisalt Buffer (0.05% digitonin, 20 mM HEPES, pH 7.5, 300 mM NaCl, 0.5 mM spermidine, 1× protease inhibitor cocktail). The cells were incubated with the protein A‐Tn5 transposome at 25°C for 1 h and subsequently washed three times with Dig‐Hisalt Buffer to remove any unbound protein A‐Tn5. The cells were then resuspended in 50 µL of Tagmentation buffer (10 mM MgCl2 in Dig‐Hisalt Buffer) and incubated at 37°C for 1 h. Tagmentation was halted by the addition of 1 µL of 10% SDS, followed by incubation at 55°C for 10 min. Finally, the DNA fragments were extracted using phenol‒chloroform. The following primary antibodies were used: anti‐Lamin B1 (Abcam, ab133741), anti‐H3K27ac (Abcam, ab177178), anti‐BRD4 (Abcam, ab128874), anti‐POL II (Abcam, ab26721), anti‐H3K9me2 (Abcam, ab32521), and anti‐H3K9me3 (Abcam, ab8898).

### 2.18 CUT&Tag‐qPCR

Following tagmentation, DNA fragments were extracted using phenol‒chloroform and precipitated with ethanol. The purified DNA was resuspended in nuclease‐free water. The enrichment of specific genomic regions was quantified by qPCR. Primer pairs were designed to amplify SLC7A11 enhancer regions. qPCRs were set up using a commercial SYBR Green qPCR Master Mix. All qPCRs were performed in technical triplicates. The relative enrichment was calculated using the 2^^−ΔΔCt^ method and normalized to the signal from the IgG control. The primers for the SLC7A11 enhancer were as follows: enhancer‐1 forward (5'→3') TTCCACTTTCGGGGAAAGCA, reverse (5'→3') GCAACAAAGATCGGGACTGC, enhancer‐2 forward (5'→3') TCTTGCTCTGTGCGTGTCTT, reverse (5'→3') AGCACAGCGTGATTTCCTGA.

### 2.19 CUT&Tag Sequencing and Data Processing

The libraries were sequenced using an Illumina NovaSeq 6000 platform at Novogene Science and Technology Co., Ltd. (Beijing, China), generating 150 bp paired‐end reads. Initially, raw reads were filtered and trimmed to eliminate sequencing adapters and low‐quality reads using TrimGalore (v0.6.6) with the parameters ‐q 20 –phred33 –stringency 3. The clean reads were then mapped to the human genome reference hg38 with Bowtie2 (v2.5.1) using the default parameter. Peak calling was conducted with MACS2 (v2.1.1) using the parameters ‐q 0.05 –call‐summits –nomodel –shift ‐100 –extsize 200 –keep‐dup all. Heatmaps were created using the computeMatrix and plotHeatmap commands in deepTools (v2.3.6.0).

### 2.20 Super‐Enhancer Identification

Enhancers were predicted from the peak regions of H3K27ac, BRD4, and POL II that are located at least 2 kb away from annotated transcription start sites (TSSs). Constituent enhancers within a 12.5 kb radius were combined to identify super‐enhancers (SEs) using the ROSE algorithm version 2, developed by the Young laboratory. Enhancers within this 12.5 kb region were stitched together to create a single entity representing a genomic region. The stitched enhancers, along with individual enhancers that did not have neighboring enhancers within the 12.5 kb range, were ranked based on their peak signal levels in the genomic region. By definition, stitched or individual enhancers with signal levels exceeding those of a slope of 1 on the intensity distribution plot were classified as SEs, while the remaining enhancers were designated typical enhancers (TEs). The annotation of TEs and SEs was carried out using the annotatePeaks.pl script in Homer (v4.11.1) with default parameters.

### 2.21 Chromatin State Classification

For the ChromHMM analysis, we first binarized the data at a resolution of 200 base pairs using the BinarizeBed command in ChromHMM (v.1.25). Following the manual instructions, we created an input table named the cell mark file, which consisted of three tab‐delimited columns. We then executed the BinarizeBam command with the parameters “‐gzip ‐b 200 ‐f 0 ‐g 0 ‐p 0.0001” to binarize the CUT&Tag data. Subsequently, we developed the chromatin state model for MSCs using the LearnModel command from ChromHMM (v.1.25). To train the model with the processed input data, we utilized the LearnModel command with the options “–gzip ‐d 0.001 ‐color 0,0,255 ‐p 5 ‐holdcolumnorder ‐splitrows 7.”

### 2.22 LAD Detection

The enriched domain detector (EDD, v.1.1.19) was developed to identify LADs. We employed the parameters “–bin‐size 20 –gap‐penalty 0.5 ‐p 20” to calculate the LaminB1‐enriched signal relative to the IgG sample using EDD. The LaminB1 log2‐fold‐change ratio against the IgG sample was then used to generate bigwig files, which can be visualized with the Integrative Genomics Viewer (IGV).

### 2.23 Flow Cytometry

MSCs were plated in 12‐well plates 24 hr prior to treatment. Following incubation with drug‐supplemented or control media, adherent cells were trypsinized, pelleted by centrifugation, and resuspended in ice‐cold PBS containing 1 µg/mL propidium iodide (PI) (YEASEN, 40710ES03) or 1:1000 tetramethylrhodamine, ethyl ester (TMRE) (Beyotime, C2001S). Dead MSCs were quantified via flow cytometry (BD FACSCelesta cytometer), measuring PI‐positive populations. The mitochondrial membrane potential of MSCs was quantified by measuring the mean fluorescence intensity of TMRE staining, with data analyzed in FlowJo v10.

### 2.24 NADP^+^ and NADPH Measurements

Intracellular levels of NADPH and total NADP (NADPH + NADP^+^) were measured using the NADP^+^/NADPH assay kit (Beyotime, S0179) following the manufacturer's instructions. In brief, cells in six‐well plates were lysed in 200 µl of extraction buffer, and the lysates were collected into 1.7‐ml microtubes and centrifuged at 12,000 g and 4°C for 10 min. The supernatant from each tube was divided into two 100 µl aliquots. For NADPH measurement, one aliquot was incubated at 60°C for 30 min to degrade NADP^+^ without affecting NADPH, followed by cooling on ice. The second 100 µl aliquot was reserved for total NADP^+^ measurement. For analysis, 50 µl aliquots from all 100 µl cell supernatant aliquots (with and without heating) were added to a 96‐well plate and mixed with 100 µl of G6PDH working buffer. After incubation in the dark at 37°C for 10 min, 10 µl of chromogenic solution was added to each well containing the mixture. The absorbance was measured at 450 nm after a subsequent incubation at 37°C for 30 min using a microplate reader. The concentration of NADP^+^ was then calculated by subtracting NADPH from the total NADP.

### 2.25 Cystine Uptake Assay

Cystine uptake levels in MSCs were assessed using the Cystine Uptake Assay Kit (Dojindo, UP05). The solutions for cystine uptake, probe, and reducing agent (RA) were prepared following the provided guidelines. MSCs were plated in 96‐well plates and incubated overnight at 37°C in a 5% CO2 environment. The next day, the supernatant was carefully removed, and the MSCs were washed three times with warmed cystine‐free serum‐free medium (Gibco, 21013024). Then, the MSCs were incubated with 200 µL of warmed cystine‐free serum‐free medium for 5 min at 37°C in a 5% CO2 incubator. After discarding the supernatant, 200 µL of either the warmed CA uptake solution or cystine‐free serum‐free medium (for blank wells) was added to the cells, which were then incubated for 30 min at 37°C and 5% CO2. The supernatant was again removed, and the cells were rinsed three times with 200 µL of chilled PBS. Subsequently, 50 µL of methanol was added to each well. The working solution was then prepared according to the specified instructions, and 200 µL of this solution was added to each well for a 30‐min incubation at 37°C in a 5% CO2 incubator. Finally, the values obtained from the sample wells were subtracted from those of the blank wells, and the MSC uptake of cystine was captured using images captured using a Zeiss LSM 880 confocal microscope.

### 2.26 Oxygen Consumption Rate (OCR) Measurement

Mitochondrial respiration was assessed using the Seahorse XF Mito Stress Kit (Seahorse, 103015–100) as previously described with modification [28]. MSCs (10^4^/well) were plated in XF96 plates, treated per experimental design, and analyzed on an XFe96 instrument (Agilent). Sequential injection of mitochondrial modulators (1 µM oligomycin, 1 µM FCCP, 150 nM rotenone) enabled OCR measurement. Data normalized to protein content with basal OCR = (pre‐oligomycin OCR)—(post‐rotenone OCR), Maximal OCR = (post‐FCCP OCR)—(post‐rotenone OCR).

### 2.27 ATP Measurement Assay

Cellular ATP was quantified with a commercial assay kit (Beyotime, S0026). MSC lysates were centrifuged (12,000 ×g, 5 min, 4°C), and 20 µL supernatant aliquots were mixed with 180 µL ATP detection reagent in 96‐well plates. Luminescence (BIOTEK Synergy HTX) was recorded as relative light units normalized to total protein.

#### 2.27.1 Coimmunoprecipitation (Co‐IP)

MSC lysates were prepared using cell lysis buffer for Western blotting and IP (Beyotime, P0013). Magnetic bead‐based IP was performed with Dynabeads Protein G (Invitrogen, 10007D) per the manufacturer's protocol. Anti‐actin antibody (Abcam ab227387) was conjugated to beads in binding buffer (10 min, RT, rotation). After magnetic clearance and washing, lysates were incubated with antibody‐bead complexes (10 min, RT). The Dynabeads‐antibody complex mixture was washed with washing buffer, and the proteins were separated using a standard SDS‒PAGE system.

### 2.28 Molecular Docking

The structural data of SLC7A11 (PDB IDs: 7P9V, 7EPZ, 7P9U) were obtained from the RCSB Protein Data Bank (http://www.rcsb.org/). Using PyMOL (version 2.5.5), cocrystallized ligands and heteroatoms were removed, and water molecules were eliminated. Hydrogen atoms were incorporated into the protein structures using AutoDockTools (version 1.5.7). Molecular structures of FDA‐approved drugs in mol2 format were acquired from the ZINC database (https://zinc.docking.org/) and converted to PDB format using Open Babel (version 3.3.1), during which hydrogen atoms were added and partial charges were assigned. Docking simulations were carried out with AutoDock Vina (version 1.1.2), and molecules were prioritized based on the lowest predicted binding affinity. The interactions between SLC7A11 and the molecules during docking were examined using the protein‒ligand interaction profiler (plip‐tool.biotec.tu‐dresden.de), and the resulting SLC7A11‐drug complexes were visualized using PyMOL.

### 2.29 Molecular Dynamics Simulation

Molecular dynamics simulations were conducted using the AmberTools 20 package. The SLC7A11 structures (PDB IDs: 7P9V) were initially prepared by removing crystallographic water and ligands with PyMOL, followed by the reconstruction of missing atoms using SPDBV (version 4.1.0). The protein and the cocrystallized compound were parameterized with the AMBER ff19SB and GAFF force fields, respectively. The system was solvated in a truncated octahedral box of OPC water molecules with a 10 Å buffer. Following charge neutralization, the system underwent a three‐stage energy minimization process (consisting of 5,000, 5,000, and 20,000 steps, respectively). Subsequently, the system was heated to 300 K over 20 ps in the NVT ensemble using a Langevin thermostat. This was followed by a 200 ps equilibration in the NPT ensemble (300 K, Langevin thermostat) and a subsequent 1.0 ns equilibration in the NVT ensemble (300 K, Berendsen thermostat). Finally, a production simulation of 40 ns was performed under NVT conditions at 300 K utilizing a Berendsen thermostat without restraints. Trajectory analysis was carried out using the CPPTRAJ module.

### 2.30 Statistics and Reproducibility

GraphPad Prism 8.0 was used for all statistical analyses. Unpaired Student's t tests were applied for comparisons between two groups. For comparisons among three or more groups, one‐way analysis of variance (ANOVA) followed by Tukey's post hoc test was used. Mitochondrial morphology proportions across groups were analyzed using two‐way ANOVA, with Tukey's multiple comparisons test for multi‐group contrasts and Sidak's test for pairwise comparisons. The relationship between LMNB1 expression in MSCs and ARS intensity was assessed by linear regression analysis. A *p* value < 0.05 was considered statistically significant. No statistical methods were used to predetermine the sample size. The data distribution was assumed to be normal without formal testing. All data were included in the analyses, with no exclusions. For in vitro and in vivo experiments, samples or animals were assigned to treatment groups by simple randomization. Investigators were not blinded to group allocation during experiments or outcome assessment.

## 3 Results

### 3.1 Age‐related LMNB1 Deficiency in MSCs Impairs the Osteogenesis of SOP by Triggering Actin Cytoskeleton Collapse

The downregulation of LMNB1 expression is a significant age‐related hallmark that is widely involved in the development of various degenerative disorders [29, 30]. To investigate the role of LMNB1 in the impaired osteogenesis of SOP, we generated *LMNB1^△Prx1^* mice with conditional LMNB1 knockout in limb mesenchyme‐derived cells (Extended Data Figure 1A–D), as MSCs are the primary source of osteoblasts. *LMNB1^△Prx1^* mice exhibited decreased bone mass, as evidenced by micro‐CT analysis showing significant reductions in bone volume/total volume (BV/TV), trabecular thickness (Tb.Th), trabecular number (Tb.N), cortical thickness (Ct.Th) and cortical bone volume (Ct.BV), along with increased trabecular spacing (Tb.Sp), compared to *LMNB1^fl/fl^* littermates (Figure 1A). Consistently, femur sections from *LMNB1^△Prx1^* mice showed reduced bone trabecular area and diminished OSX^+^ cell numbers (Figure 1B). Calvarial defect models showed impaired capacity for bone repair in *LMNB1^△Prx1^* mice at 8 weeks post‐surgery compared to controls (Figure 1C). In vitro osteogenic assays showed decreased ARS and ALP staining intensity, along with reduced expression of osteogenic markers, including RUNX2, OSX and COL1, which indicated the impaired osteogenic differentiation potential of *LMNB1^△Prx1^* MSCs (Figure 1D, E and Extended Data Figure 1E). These findings collectively suggest that LMNB1 deficiency in MSCs impairs osteogenesis.

**FIGURE 1:**
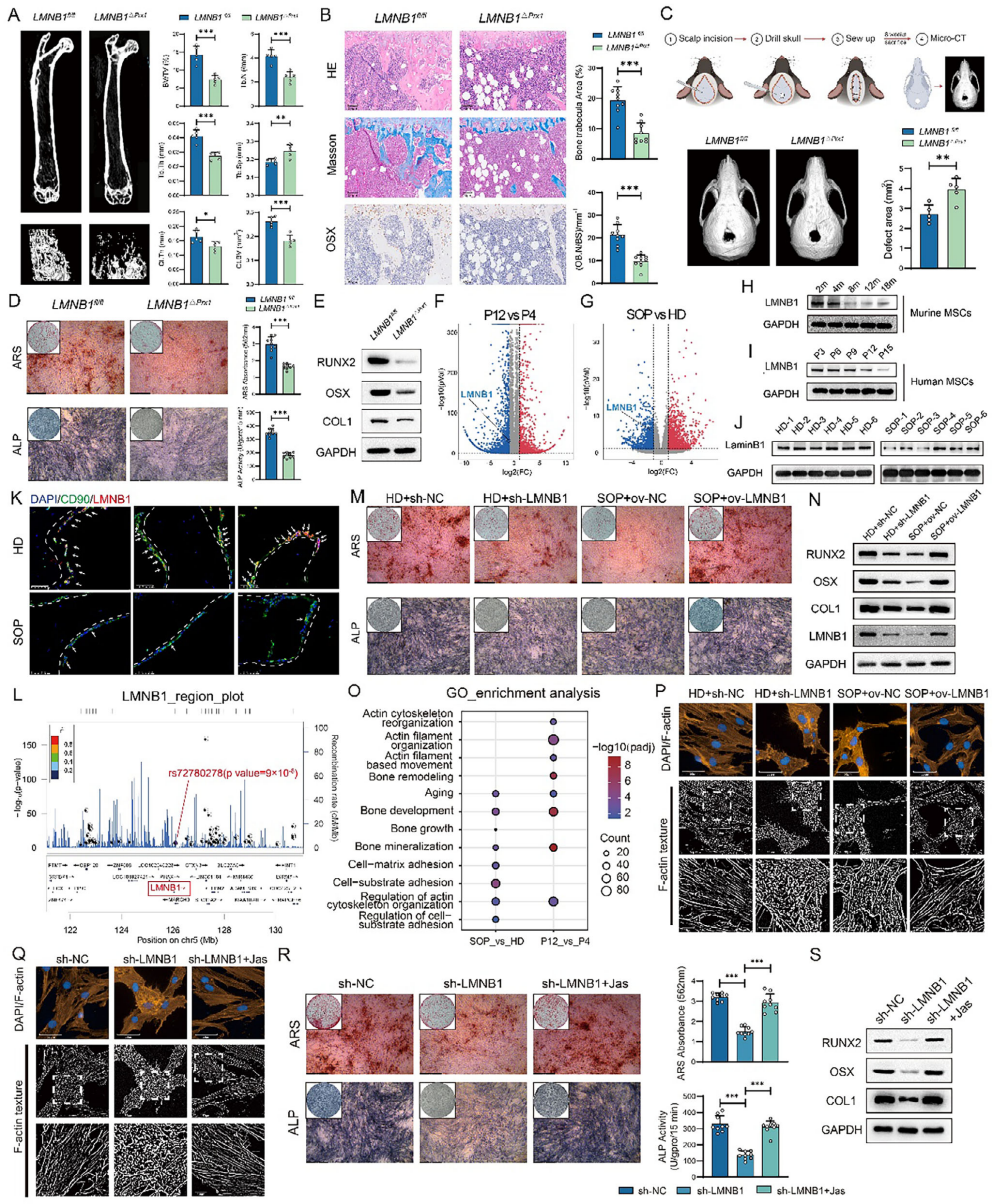
Age‐related LMNB1 deficiency in MSCs impairs the osteogenesis of SOP by triggering actin cytoskeleton collapse. (A) Representative micro‐CT images of femoral trabecular bone from *LMNB1^fl/fl^* and *LMNB1^△Prx1^* mice, with 3D reconstruction, and quantitative analysis of trabecular and cortical bone parameters in the femurs (n = 5 mice per group). (B) Histological sections of tibias from *LMNB1^fl/fl^* or *LMNB1^△Prx1^* mice stained with H&E, Masson's trichrome, and OSX immunohistochemistry and quantitative analysis of Masson‐positive area and osteoblast number per bone surface (OB.N/BS). (C) Schematic diagram of the calvarial defect model (top). Representative micro‐CT images showing calvarial defect healing in *LMNB1^fl/fl^* and *LMNB1^△Prx1^* mice and quantitative analysis of the calvarial defect areas (n = 5 mice per group). (D) ARS and ALP staining of MSCs from *LMNB1^fl/fl^* or *LMNB1^△Prx1^* mice during osteogenic differentiation and quantitative analysis of ARS intensity and ALP activity. (E) Western blot analysis of RUNX2, OSX and COL I expression in MSCs from *LMNB1^fl/fl^* or *LMNB1^△Prx1^* mice during osteogenic differentiation. (F) Volcano plot of RNA‐seq data comparing MSCs at passage 12 and MSCs at passage 4 during osteogenic differentiation. (G) Volcano plot of RNA‐seq data comparing SOP‐MSCs and HD‐MSCs during osteogenic differentiation. (H) Western blot analysis of LMNB1 expression in MSCs extracted from mice aged 2, 4, 8, 12, and 18 months during osteogenic differentiation. (I) Western blot analysis of LMNB1 expression in MSCs at passages 3, 6, 9, 12 and 15 during osteogenic differentiation. (J) Western blot analysis of LMNB1 expression in HD‐MSCs and SOP‐MSCs during osteogenic differentiation. (K) Representative immunofluorescence images showing LMNB1 expression in MSCs of bone tissues from HD subjects or SOP patients. (L) Regional association plot of the LMNB1 locus for osteoporosis (p value = 9×10^‐8). (M) ARS and ALP staining of osteogenic differentiating HD‐MSCs+sh‐NC, HD‐MSCs +sh‐LMNB1, SOP‐MSCs+ov‐NC and SOP‐MSCs+ov‐LMNB1. (N) Western blot analysis of RUNX2, OSX, COL I and LMNB1 expression in HD‐MSCs+sh‐NC, HD‐MSCs+sh‐LMNB1, SOP‐MSCs+ov‐NC and SOP‐MSCs+ov‐LMNB1 during osteogenic differentiation. (O) GO enrichment analysis of DEGs from two RNA‐seq datasets (SOP‐MSCs vs HD‐MSCs, P12 MSCs vs P4 MSCs). (P) Representative TRITC‐Phalloidin immunofluorescence images captured by a high‐content analysis system showing the actin network texture of HD‐MSCs+sh‐NC, HD‐MSCs+sh‐LMNB1, SOP‐MSCs+ov‐NC and SOP‐MSCs+ov‐LMNB1. (Q) Representative TRITC‐Phalloidin immunofluorescence images captured by a high‐content analysis system showing the actin network texture of MSCs treated with sh‐NC, sh‐LMNB1 or sh‐LMNB1+ jasplakinolide (10 nM). (R) ARS and ALP staining of MSCs treated with sh‐NC, sh‐LMNB1 or sh‐LMNB1+ jasplakinolide during osteogenic differentiation and quantitative analysis of ARS intensity and ALP activity. (S) Western blot analysis of RUNX2, OSX and COL I expression in MSCs treated with sh‐NC, sh‐LMNB1 or sh‐LMNB1+ jasplakinolide during osteogenic differentiation. The data are presented as the means ± standard deviations (SD), n = 9 (unless stated), ^*^ *p* < 0.05, ^**^ *p* < 0.01, ^***^ *p* < 0.005.

RNA sequencing data determined LMNB1 downregulation in senile human MSCs at passage 12 compared to juvenile MSCs at passage 4, as well as LMNB1 downregulation in SOP‐MSCs compared to HD‐MSCs (Figure 1F, G). At the protein level, LMNB1 expression continuously decreased as the age of the MSC‐derived mice increased (Figure 1H and Extended Data Figure 1F) and as the passage numbers of human MSCs increased (Figure 1I and Extended Data Figure 1G). Notably, LMNB1 expression was decreased in SOP‐MSCs compared to HD‐MSCs, with a positive correlation between the LMNB1 expression levels of MSCs and the ARS intensity across multiple donors (Figure 1J and Extended Data Figures 1H, I). LMNB1 downregulation in SOP‐MSCs was also confirmed in bone tissue by immunofluorescence (Figure 1K). In addition, GWAS data from the NHGRI indicated an association between LMNB1 SNPs and osteoporosis susceptibility (p = 9×10^−^^8^) (Figure 1L). In vitro osteogenic assays demonstrated that inhibiting LMNB1 expression impeded the osteogenic differentiation of HD‐MSCs, and overexpressing LMNB1 in SOP‐MSCs rescued their osteogenic differentiation to levels comparable to those of HD‐MSCs (Figure 1M, N and Extended Data Figure 1J, K). These data reveal that age‐related LMNB1 deficiency impairs the osteogenesis of SOP‐MSCs.

Integrated enrichment analysis of the differentially expressed genes of the two abovementioned RNA sequencing datasets was performed to explore the mechanism by which LMNB1 regulates MSC osteogenic differentiation. In addition to those terms related to aging and osteogenesis, several terms about cytoskeleton‐related processes were significantly enriched (Figure 1O), indicating that LMNB1 deficiency impaired SOP‐MSC osteogenesis by affecting the actin cytoskeleton. High‐content fluorescence imaging with quantitative texture analysis showed that SOP‐MSCs displayed disorganized actin networks with reduced bundle formation, decreased SER Ridge and anisotropy of actin compared to HD‐MSCs. This actin cytoskeleton collapse was mimicked in HD‐MSCs by LMNB1 knockdown and rescued in SOP‐MSCs by LMNB1 overexpression (Figure 1P and Extended Data Figure 2A). Moreover, treatment with the actin‐stabilizing agent jasplakinolide (Jas) prevented LMNB1 knockdown‐induced actin cytoskeleton collapse and the subsequent impaired osteogenesis of HD‐MSCs (Figure 1Q–S and Extended Data Figure 2B, C). Similar results were observed in MSCs from *LMNB1^△Prx1^* mice (Extended Data Figure 2D–F). All the data above demonstrate that age‐related LMNB1 deficiency in MSCs leads to impaired osteogenesis of SOP via actin cytoskeleton collapse.

**FIGURE 2:**
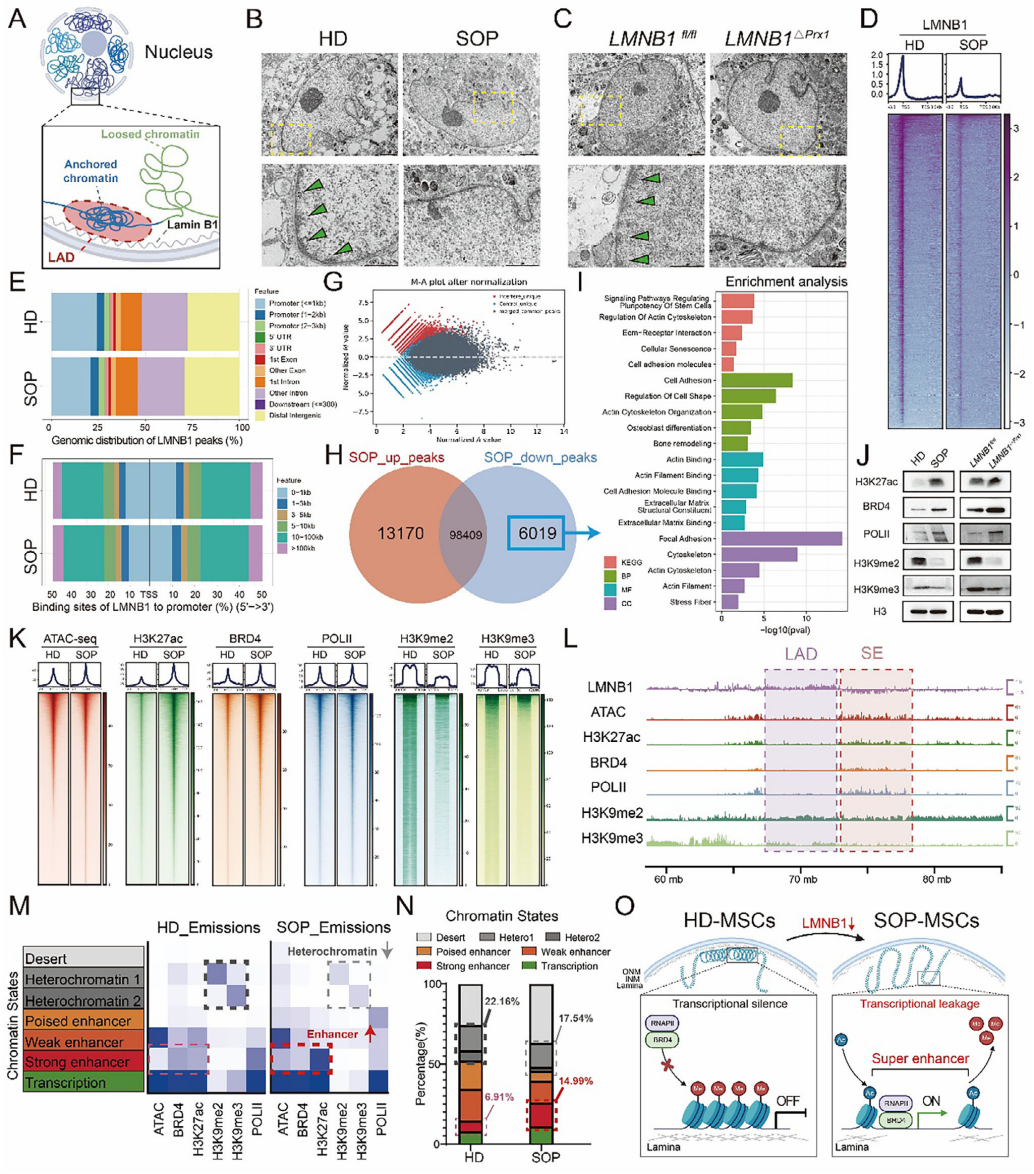
LMNB1 deficiency promotes SE formation to drive aberrant expression of actin cytoskeleton collapse‐related molecules via LAD detachment. (A) Schematic diagram of the anchored chromatin in LADs and loose chromatin detached from LADs. (B) Representative transmission electron microscopy images showing chromatin anchoring to the nuclear periphery of HD‐MSCs and SOP‐MSCs. (C) Representative transmission electron microscopy images showing chromatin anchoring to the nuclear periphery of MSCs from *LMNB1^fl/fl^* or *LMNB1^△Prx1^* mice. (D) CUT&Tag profile heatmap showing genomic LMNB1 abundance in HD‐MSCs and SOP‐MSCs. (E) Genomic distribution of LMNB1 peaks across the genome in HD‐MSCs and SOP‐MSCs. (F) Positional relationship between LMNB1 peaks and promoters in HD‐MSCs and SOP‐MSCs. (G) MAnorm analysis showing differential LMNB1 peaks between HD‐MSCs and SOP‐MSCs. (H) Venn diagram showing the numbers of differential and common LMNB1 peaks. (I) KEGG and GO enrichment analysis of the genes with downregulated LMNB1 peaks in SOP‐MSCs. (J) Western blot analysis of chromatin proteins (H3K27ac, BRD4, POL II, H3K9me2, and H3K9me3) in HD‐MSCs, SOP‐MSCs, and MSCs from *LMNB1^fl/fl^* or *LMNB1^△Prx1^* mice during osteogenic differentiation. (K) Profile heatmap showing general genomic levels of ATAC‐seq and CUT&Tag (H3K27ac, BRD4, POL II, H3K9me2 and H3K9me3) in HD‐MSCs and SOP‐MSCs. (L) Signal traces of ATAC‐seq and CUT&Tag (LMNB1, H3K27ac, BRD4, POL II, H3K9me2 and H3K9me3) data. The shadow in purple shows the LAD region, and the shadow in red shows the SE region. (M) ChromHMM‐based chromatin state classification using ATAC‐seq and CUT&Tag data (H3K27ac, BRD4, POL II, H3K9me2 and H3K9me3) in HD‐MSCs and SOP‐MSCs. (N) Bar plot showing the percentages of the chromatin state in HD‐MSCs and SOP‐MSCs. (O) Schematic diagram of SE‐mediated transcriptional leakage in SOP‐MSCs at the LAD detachment regions. The data are presented as the means ± standard deviations (SD), n = 9 (unless specifically stated), ^*^ *p* < 0.05, ^**^ *p* < 0.01, ^***^ *p* < 0.005.

### 3.2 LMNB1 Deficiency Promotes SE Formation to Drive Aberrant Expression of Actin Cytoskeleton Collapse‐Related Molecules

LMNB1 deficiency can lead to chromatin detachment and decompaction from the nuclear membrane, therefore resulting in alterations in the gene expression profile (Figure 2A). Transmission electron microscopy (TEM) showed reduced chromatin anchoring at the nuclear periphery in both SOP‐MSCs and *LMNB1^△Prx1^* MSCs compared with their respective controls (Figure 2B, C). CUT&Tag analysis confirmed global reduced binding of LMNB1 to chromatin in SOP‐MSCs (Figure 2D), and ChIPseeker and MAnorm analyses identified the differential LMNB1 binding patterns of SOP‐MSCs compared to HD‐MSCs (Figure 2E–G). The identified genes with reduced LMNB1 binding peaks in SOPs were enriched in multiple terms related to the actin cytoskeleton (Figure 2H, I). We postulated that LMNB1 deficiency might cause LAD region detachment and result in heterochromatin loss and increased chromatin accessibility, therefore driving actin cytoskeleton collapse‐related gene expression.

SEs, functional clusters of multiple typical enhancers that efficiently drive gene expression, are preferentially established in genomic regions characterized by increased chromatin accessibility [31]. Western blot analysis of chromatin proteins revealed decreased heterochromatin markers (H3K9me3 and H3K9me2) and elevated enhancer markers (H3K27ac, BRD4 and POL II) in SOP‐MSCs and *LMNB1^△Prx1^* MSCs compared with their respective controls (Figure 2J and Extended Data Figure 3A). CUT&Tag profiling confirmed a global reduction in genomic H3K9me3 and H3K9me2 and increased H3K27ac, BRD4 and POL II. in SOP‐MSCs, and ATAC‐seq confirmed increased chromatin accessibility in these cells (Figure 2K). At LAD‐detached regions, chromatin decompaction enabled SE formation accompanied by heterochromatin loss (Figure 2L, Extended data Figure 3B, Table S3). Notably, both CUT&Tag and ATAC‐seq analyses revealed significant differences in the genomic distribution and positional relation with promoters between HD‐MSCs and SOP‐MSCs (Extended Data Figure 3C, D). ChromHMM‐based chromatin state classification (7 states) demonstrated reduced heterochromatin regions and increased stronger enhancer regions in SOP‐MSCs (Figure 2M, N). These results indicate that LMNB1 deficiency disrupts chromatin anchoring and LAD region formation, which promotes SE formation to drive the aberrant expression of actin cytoskeleton collapse‐related molecules in SOP‐MSCs (Figure 2O, Table S4).

**FIGURE 3:**
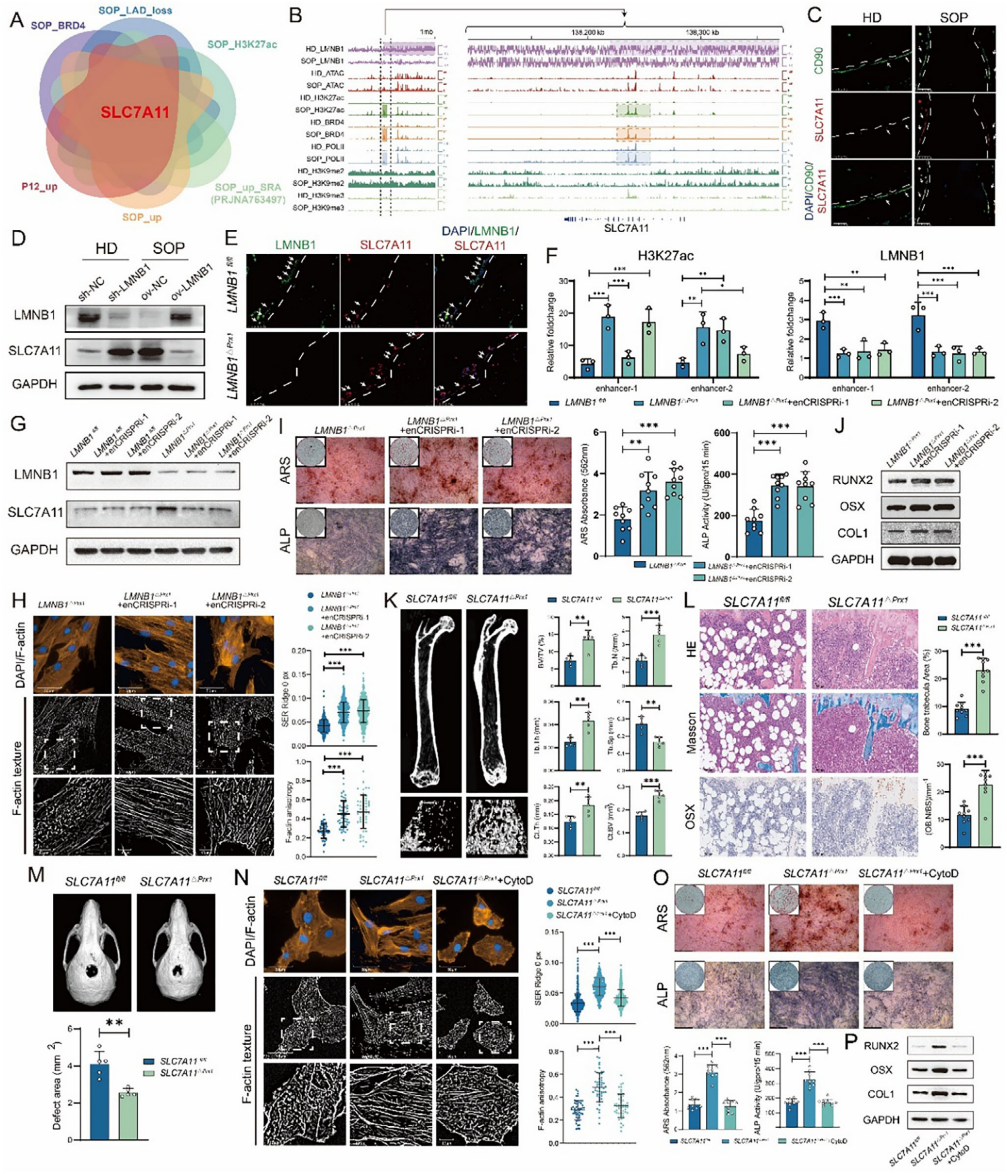
SLC7A11 as the pivotal molecule in LMNB1 deficiency‐mediated dysfunction of SOP‐MSCs, (A) Venn diagram identifying transcriptional leaked genes by the overlap of genes upregulated in P12 MSCs and SOP‐MSCs and related to LAD‐detached and SE regions in SOP‐MSCs, and SLC7A11 was identified as the only pivotal gene. (B) SLC7A11 signal traces of ATAC‐seq and CUT&Tag (LMNB1, H3K27ac, BRD4, POL II, H3K9me2 and H3K9me3) data. The shadow in purple shows the LAD region, and the shadows in green, orange and blue show newly formed SE in LAD‐detachment regions. (C) Representative immunofluorescence images showing SLC7A11 expression in bone tissues from HD subjects or SOP patients. (D) Western blot analysis of LMNB1 and SLC7A11 expression in HD‐MSCs+sh‐NC, HD‐MSCs+sh‐LMNB1, SOP‐MSCs+ov‐NC and SOP‐MSCs+ov‐LMNB1 during osteogenic differentiation. (E) Representative immunofluorescence images showing LMNB1 and SLC7A11 expression in the tibia from *LMNB1^fl/fl^* or *LMNB1^△Prx1^* mice. (F) H3K27ac and LMNB1 CUT&Tag‐qPCR analysis of *LMNB1^fl/fl^* MSCs*, LMNB1^△Prx1^* MSCs, *LMNB1^△Prx1^* MSCs+enCRISPRi‐1 and *LMNB1^△Prx1^* MSCs+enCRISPRi‐2 at enhancer‐1 and enhancer‐2. (G) Western blot analysis of LMNB1 and SLC7A11 expression in *LMNB1^fl/fl^* MSCs*, LMNB1^fl/fl^* MSCs+enCRISPRi‐1 and *LMNB1^fl/fl^* MSCs+enCRISPRi‐2, *LMNB1^△Prx1^* MSCs, *LMNB1^△Prx1^* MSCs+enCRISPRi‐1 and *LMNB1^△Prx1^* MSCs+enCRISPRi‐2 during osteogenic differentiation. (H) Representative TRITC‐Phalloidin immunofluorescence images captured by a high‐content analysis system showing the actin network texture of *LMNB1^△Prx1^* MSCs, *LMNB1^△Prx1^* MSCs+enCRISPRi‐1 and *LMNB1^△Prx1^* MSCs+enCRISPRi‐2 and actin morphology quantitative analysis of actin SER Ridge (n = 402–509 cells) and anisotropy (n = 50 cells). (I) ARS and ALP staining of *LMNB1^△Prx1^* MSCs, *LMNB1^△Prx1^* MSCs+ enCRISPRi‐1, *LMNB1^△Prx1^* MSCs+ enCRISPRi‐2, and quantitative analysis of ARS intensity and ALP activity. (J) Western blot analysis of RUNX2, OSX and COL I expression in *LMNB1^△Prx1^* MSCs, *LMNB1^△Prx1^* MSCs+enCRISPRi‐1 and *LMNB1^△Prx1^* MSCs+enCRISPRi‐2 during osteogenic differentiation. (K) Representative micro‐CT images of femoral trabecular bone from 18‐month *SLC7A11^fl/fl^* and *SLC7A11^△Prx1^* mice, with 3D reconstruction, and quantitative analysis of trabecular and cortical bone parameters in the femurs (n = 5). (L) Histological sections of tibias from 18‐month *SLC7A11^fl/fl^* and *SLC7A11^△Prx1^* mice stained with H&E, Masson's trichrome, and OSX immunohistochemistry and quantitative analysis of Masson‐positive area and OB.N/BS (n = 5 mice per group). (M) Representative micro‐CT images showing calvarial defect healing in 18‐month *SLC7A11^fl/fl^* and *SLC7A11^△Prx1^* mice and quantitative analysis of the calvarial defect areas (n = 5 mice per group). (N) Representative TRITC‐Phalloidin immunofluorescence images captured by a high‐content analysis system showing the actin network texture of *SLC7A11^fl/fl^* MSCs, *SLC7A11^△Prx1^* MSCs or *SLC7A11^△Prx1^* MSCs+CytoD (500 nM) and actin morphology quantitative analysis of actin SER Ridge (n = 336–613 cells) and anisotropy (n = 50 cells). (O) ARS and ALP staining of *SLC7A11^fl/fl^* MSCs, *SLC7A11^△Prx1^* MSCs or *SLC7A11^△Prx1^* MSCs+ CytoD during osteogenic differentiation and quantitative analysis of ARS intensity and ALP activity. (P) Western blot analysis of RUNX2, OSX and COL I expression in *SLC7A11^fl/fl^* MSCs, *SLC7A11^△Prx1^* MSCs or *SLC7A11^△Prx1^* MSCs+CytoD during osteogenic differentiation. The data are presented as the means ± standard deviations (SD), n = 9 (unless specifically stated), ^*^ *p* < 0.05, ^**^ *p* < 0.01, ^***^ *p* < 0.005.

### 3.3 SLC7A11 As the Pivotal Molecule in LMNB1 Deficiency‐Mediated Dysfunction of SOP‐MSCs

Multiomics integration, including an external reference SRA database comparing HD‐MSCs and SOP‐MSCs (PRJNA763497), identified the sole gene, SLC7A11, which is located at LAD‐loss regions with concurrent SE formation and is transcriptionally upregulated in both senile MSCs and SOP‐MSCs (Figure 3A, Table S5). At the SLC7A11 locus, SOP‐MSCs exhibited LAD chromatin detachment, increased chromatin accessibility (ATAC‐seq), SE formation (H3K27ac/BRD4/POL II accumulation), and heterochromatin loss (H3K9me3/H3K9me2 reduction) (Figure 3B). Immunofluorescence analysis of bone tissue from SOP subjects and western blot assays showed the upregulation of SLC7A11 in SOP‐MSCs (Figure 3C, D and Extended data Figure 4A). In addition, knocking down LMNB1 in HD‐MSCs caused SOP‐MSC‐like SLC7A11 upregulation, whereas LMNB1 overexpression effectively suppressed SLC7A11 expression in SOP‐MSCs (Figure 3D). These data, as well as the immunofluorescence showing the elevated SLC7A11 expression in bone tissue of *LMNB1^△Prx1^* mice (Figure 3E), indicated the causal relationship between LMNB1 and SLC7A11.

**FIGURE 4:**
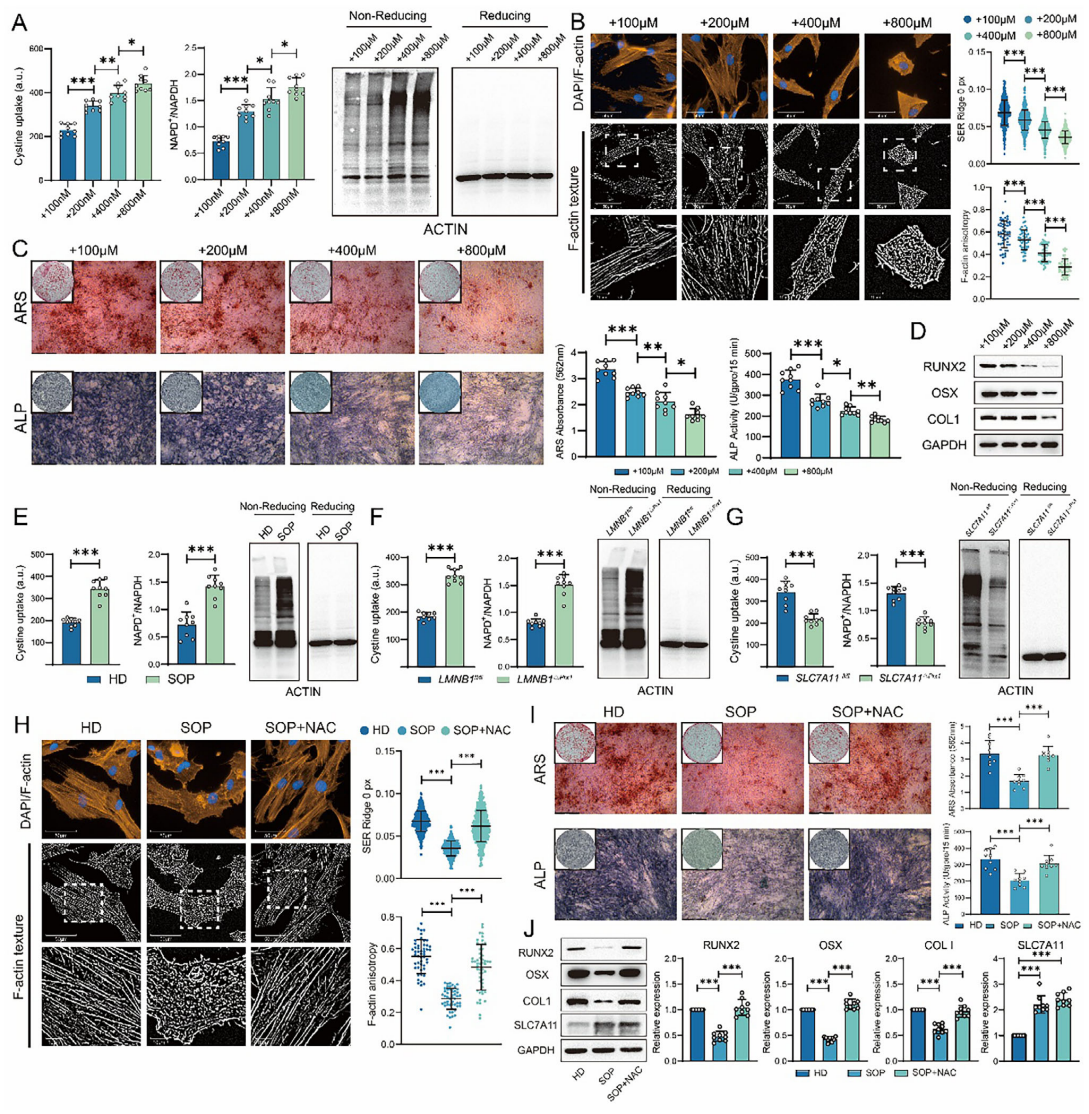
Disulfidptosis activation contributed to actin cytoskeleton collapse and impaired osteogenesis of SOP‐MSCs. (A) Cystine uptake, NADP^+^/NADPH ratios and non‐reducing and reducing western blot analysis of actin in SOP‐MSCs treated with 100 µM, 200 µM, 400 µM or 800 µM cystine. (B) Representative TRITC‐Phalloidin immunofluorescence images captured by a high‐content analysis system showing the actin network texture of SOP‐MSCs treated with 100 µM, 200 µM, 400 µM or 800 µM cystine and actin morphology quantitative analysis of the actin SER Ridge (n = 479–706 cells) and anisotropy (n = 50 cells). (C) ARS and ALP staining of SOP‐MSCs treated with 100 µM, 200 µM, 400 µM or 800 µM cystine and quantitative analysis of ARS intensity and ALP activity. (D) Western blot analysis of RUNX2, OSX and COL I expression in SOP‐MSCs treated with 100 µM, 200 µM, 400 µM or 800 µM cystine during osteogenic differentiation. (E) Cystine uptake, NADP^+^/NADPH ratios and non‐reducing and reducing western blot analysis of actin in HD‐MSCs and SOP‐MSCs. (F) Cystine uptake, NADP^+^/NADPH ratios and non‐reducing and reducing western blot analysis of actin in *LMNB1^fl/fl^* MSCs and *LMNB1^△Prx1^* MSCs. (G) Cystine uptake, NADP^+^/NADPH ratios and non‐reducing and reducing western blot analysis of Actin in *SLC7A11^fl/fl^* MSCs and *SLC7A11^△Prx1^* MSCs. (H) Representative TRITC‐Phalloidin immunofluorescence images captured by a high‐content analysis system showing the actin network texture of HD‐MSCs, SOP‐MSCs and SOP‐MSCs+NAC (2 mM) and actin morphology quantitative analysis of actin SER Ridge (n = 362–497 cells) and anisotropy (n = 50 cells). (I) ARS and ALP staining of HD‐MSCs, SOP‐MSCs and SOP‐MSCs+NAC and quantitative analysis of ARS intensity and ALP activity. (J) Western blot analysis of RUNX2, OSX, COL I and SLC7A11 expression in HD‐MSCs, SOP‐MSCs and SOP‐MSCs+NAC during osteogenic differentiation and relative quantitative analysis of RUNX2, OSX, COL I and SLC7A11 expression. The data are presented as the means ± standard deviations (SD), n = 9 (unless specifically stated), ^*^ *p* < 0.05, ^**^ *p* < 0.01, ^***^ *p* < 0.005.

To further investigate the effect of LMNB1‐mediated SEs in SLC7A11 upregulation, we identified two conserved enhancer loci by integrating the ChIP‐seq data and ATAC‐seq data of multiple osteogenic progenitor cells and then designed a CRISPR‐Cas9 system consisting of dCas9‐KRAB and sgRNA‐MS2‐SV40‐MCP‐LSD1 (enCRISPRi hereafter) to inhibit the identified SLC7A11 enhancers by suppressing histone modification (Extended Data Figure 4B, C). CUT&Tag‐qPCR analysis showed that *LMNB1^△Prx1^* MSCs recapitulated elevated H3K27ac and reduced LMNB1 levels at SLC7A11 enhancer regions in *LMNB1^△Prx1^* MSCs, and enCRISPRi decreased the H3K27ac level but did not affect the LMNB1 level (Figure 3F). In addition, enCRISPRi inhibited the aberrant SLC7A11 upregulation of MSCs derived from *LMNB1^△Prx1^* mice (Figure 3G, Extended Data Figure 4D). While a minor residual LMNB1 signal was observed at the SLC7A11 enhancer region in *LMNB1* ^△Prx1^ MSCs and enCRISPRi treated MSCs, this likely reflects a combination of the inherent background noise typical of antibody‐based chromatin assays and slight mosaicism from incomplete *Cre‐loxP* recombination. Nevertheless, the reduction in LMNB1 binding was profound and functionally significant. Notably, enCRISPRi rescued actin cytoskeleton collapse and then restored the osteogenic capacity of *LMNB1^△Prx1^* MSCs (Figure 3H–J, Extended Data Figure 4E).

We then successfully generated *SLC7A11^△Prx1^* mice and raised them to 18 months of age to confirm the role of SLC7A11 in SOP (Extended Data Figure 4F–I). Micro‐CT analysis showed significant bone loss in 18‐month‐old *SLC7A11^fl/fl^* mice rather than *SLC7A11^△Prx1^* mice (Figure 3K), and histological staining revealed reduced trabecular bones and decreased OSX^+^ cell numbers in control mice, contrasting with preserved bone architecture in 18‐month‐old *SLC7A11^△Prx1^* mice (Figure 3L). Cranial defect models demonstrated superior bone repair capacity in *SLC7A11^△Prx1^* mice compared to controls (Figure 3 M). In addition, actin cytoskeleton collapse and impaired osteogenesis were both restored in MSCs derived from 18‐month‐old *SLC7A11^△Prx1^* mice, and this effect was eliminated by treatment with the actin depolymerization agent cytochalasin D (CytoD) (Figure 3N–P, Extended Data Figure 4J). These data show that LMNB1 deficiency‐driven SE promotes SLC7A11 expression to cause actin cytoskeleton collapse, which then impairs the osteogenesis of SOP.

### 3.4 Enhanced SLC7A11 Expression Causes Actin Cytoskeleton Collapse to Impair Osteogenesis by Activating Disulfidptosis in SOP‐MSCs

Previous studies demonstrated that excessive cystine uptake by SLC7A11 led to NADPH depletion and cystine accumulation, ultimately triggering disulfidptosis [16, 32, 33, 34]. In addition, disulfidptosis‐induced actin disulfide bonds disrupt cytoskeletal polymerization and stabilization, which then impairs cellular function [35]. We first investigated whether disulfidptosis‐induced actin disulfide bonds affect MSC osteogenesis. Since cystine concentration elevation exacerbates disulfidptosis in SLC7A11‐high cells, we established cystine‐free medium supplemented with graded cystine concentrations for SOP‐MSCs. As increasing cystine concentrations progressively enhanced the disulfidptosis levels of MSCs (Extended Data Figure 5A), the cystine uptake, NADP^+^/NADPH ratio and disulfide bond level of actin were consistently elevated (Figure 4A). Correspondingly, increasing cystine concentrations aggravated actin cytoskeleton collapse and gradually decreased the osteogenic potential of MSCs (Figure 4B–D, Extended Data Figure 5B). These data demonstrated that disulfidptosis is involved in the actin cytoskeleton and osteogenesis of MSCs.

**FIGURE 5:**
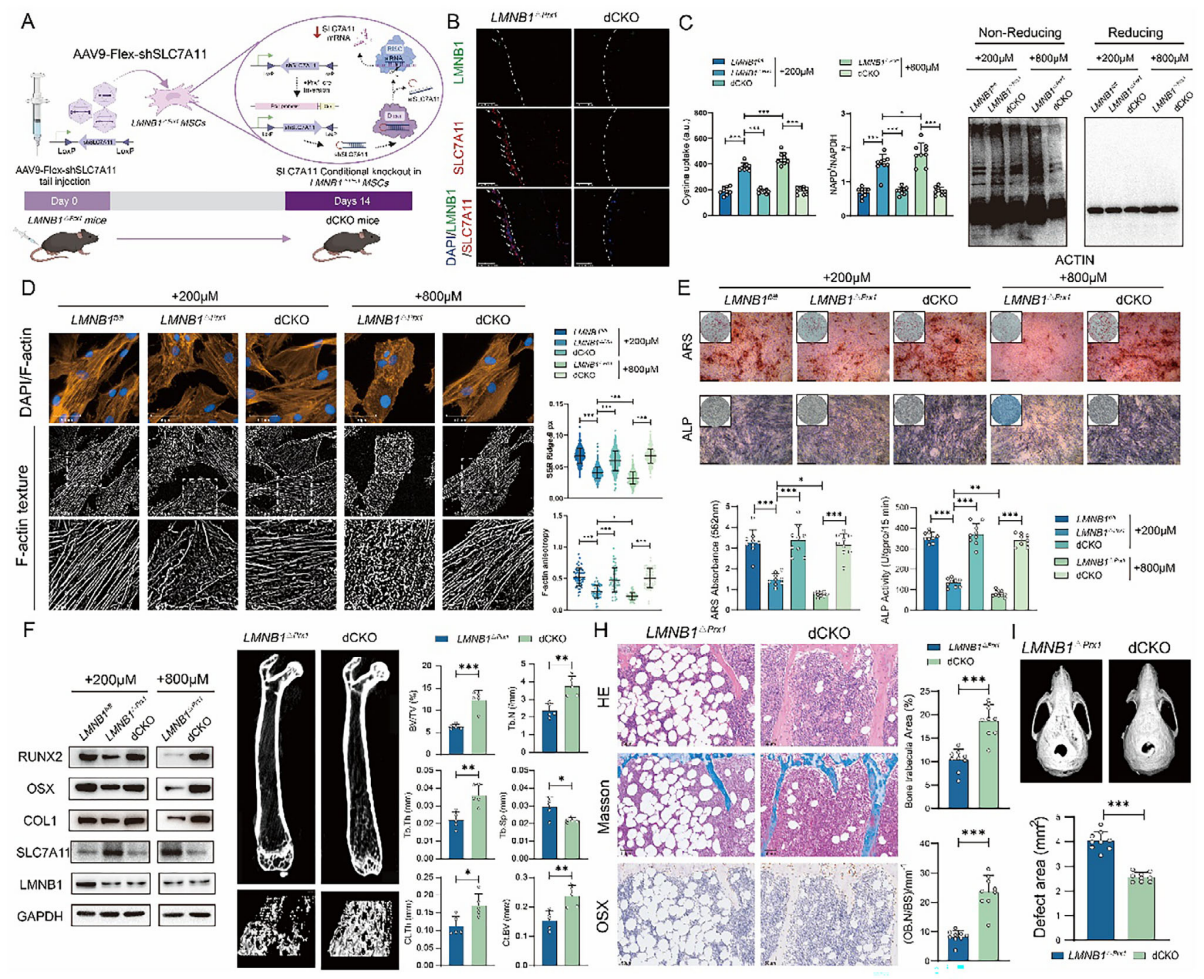
Conditional knockout of SLC7A11 in MSCs reverses disulfidptosis and restores bone mass in *LMNB1^△Prx1^* mice. (A) Schematic diagram of the dCKO construction procedure using AAV9‐Flex‐shSLC7A11. (B) Representative immunofluorescence images showing LMNB1 and SLC7A11 expression in tibias from *LMNB1^△Prx1^* or dCKO mice. (C) Cystine uptake, NADP^+^/NADPH ratios and non‐reducing and reducing western blot analysis of actin in *LMNB1^fl/fl^*, *LMNB1^△Prx1^* or dCKO MSCs cultured with 200 µM cystine and *LMNB1^△Prx1^* or dCKO MSCs cultured with 800 µM cystine. (D) Representative TRITC‐Phalloidin immunofluorescence images captured by a high‐content analysis system showing the actin network texture of *LMNB1^fl/fl^*, *LMNB1^△Prx1^* or dCKO MSCs cultured with 200 µM cystine and *LMNB1^△Prx1^* or dCKO MSCs cultured with 800 µM cystine, and actin morphology quantitative analysis of actin SER Ridge (n = 282–466 cells) and anisotropy (n = 50 cells). (E) ARS and ALP staining of *LMNB1^fl/fl^*, *LMNB1^△Prx1^* or dCKO MSCs cultured with 200 µM cystine, *LMNB1^△Prx1^* or dCKO MSCs cultured with 800 µM cystine, and quantitative analysis of ARS intensity and ALP activity. (F) Western blot analysis of RUNX2, OSX, COL I, SLC7A11 and LMNB1 expression in *LMNB1^fl/fl^*, *LMNB1^△Prx1^* or dCKO MSCs cultured with 200 µM cystine and *LMNB1^△Prx1^* or dCKO MSCs cultured with 800 µM cystine during osteogenic differentiation. (G) Representative micro‐CT images of femoral trabecular bone from *LMNB1^△Prx1^* and dCKO mice, with 3D reconstruction and quantitative analysis of trabecular and cortical bone parameters in the femurs (n = 5 mice per group). (H) Histological sections of tibias from *LMNB1^△Prx1^* and dCKO mice stained with H&E, Masson's trichrome, and OSX immunohistochemistry and quantitative analysis of Masson‐positive area and OB.N/BS (n = 5 mice per group). (I) Representative micro‐CT images showing calvarial defect healing in *LMNB1^△Prx1^* and dCKO mice and quantitative analysis of the calvarial defect areas (n = 5 mice per group). The data are presented as the means ± standard deviations (SD), n = 9 (unless specifically stated), ^*^ *p* < 0.05, ^**^ *p* < 0.01, ^***^ *p* < 0.005.

Further investigation found that SOP‐MSCs exhibited enhanced cystine uptake, an increased NADP^+^/NADPH ratio and an elevated disulfide bond of actin compared to HD‐MSCs, showing a higher disulfidptosis level (Figure 4E, Extended Data Figure 5C). Consistent results were observed in MSCs derived from *LMNB1^△Prx1^* mice compared to the corresponding controls (Figure 4F, Extended Data Figure 5D). In contrast, MSCs derived from *SLC7A11^△Prx1^* mice showed lower disulfidptosis levels, with decreased cystine uptake, NADP^+^/NADPH ratios and disulfide bond levels of actin (Figure 4G, Extended Data Figure 5E). N‐acetylcysteine (NAC), an inhibitor of disulfidptosis, successfully intercepted the disulfidptosis of SOP‐MSCs, which then effectively rectified the actin cytoskeleton collapse of SOP‐MSCs to a morphology resembling that of HD‐MSCs (Figure 4H, Extended Data Figure 5C). Furthermore, without affecting SLC7A11 expression, NAC treatment significantly restored the osteogenic capacity of SOP‐MSCs to an extent comparable to that of HD‐MSCs (Figure 4I, J), suggesting that disulfidptosis activation contributed to actin cytoskeleton collapse and impaired osteogenesis of SOP‐MSCs.

We then constructed LMNB1 and SLC7A11 double knockout (dCKO) mice by injecting AAV9‐Flex‐shSLC7A11 into *LMNB1^△Prx1^* mice, which achieved conditional knockout of SLC7A11 in *LMNB1^△Prx1^* limb mesenchyme‐derived cells (Figure 5A, B). In vitro experiments showed that with 200 µM cystine treatment (concentration equivalent to standard medium), MSCs derived from dCKO mice showed lower disulfidptosis levels manifested by decreased cystine uptake, NADP^+^/NADPH ratio and disulfide bond formation in actin compared to those from *LMNB1^△Prx1^* mice. Notably, higher cystine at a concentration of 800 µM further exacerbated the disulfidptosis of *LMNB1^△Prx1^* MSCs, while this treatment did not affect dCKO MSCs, indicating the critical role of SLC7A11 in the disulfidptosis of MSCs (Figure 5C, Extended Data Figure 5F). Consistent results were shown by the restored actin cytoskeleton microarchitecture and osteogenic differentiation capacity of dCKO MSCs, which were not affected by increases in cystine concentration (Figure 5D–F, Extended Data Figure 5G). In addition, dCKO mice had restored bone mass, as determined by micro‐CT, accompanied by increased trabecular bone area and numbers of OSX^+^ cells in bone tissue (Figure 5G, H). The cranial defect model also showed accelerated bone repair in dCKO mice (Figure 5I). The aforesaid results demonstrated that LMNB1‐mediated SLC7A11 upregulation activated disulfidptosis, therefore promoting disulfide bond formation in actin to cause cytoskeleton collapse and subsequent impaired osteogenesis of SOP‐MSCs.

### 3.5 Disulfidptosis‐Induced Disulfide Bond Formation in Actin Disrupts DRP1‐Mediated Mitochondrial Fission to Suppress Osteogenesis

Cell differentiation requires significantly increased energy provided by mitochondria, and the actin cytoskeleton is involved in cellular bioenergetic homeostasis by mediating mitochondrial morphology and function [36]. Therefore, we hypothesize that disulfidptosis‐induced disulfide bond formation in actin may affect MSC osteogenesis by regulating the energy metabolism of mitochondria. Using high‐content microscopy with machine learning, mitochondrial morphology was classified into fragmented, intermediate, and tubulated (Figure 6A). Our data revealed that senile MSCs from *SLC7A11^fl/fl^* mice at the age of 18 months had a larger amount of tubulated mitochondria but less fragmented mitochondria, which was reversed in those from *SLC7A11^△Prx1^* mice. In addition, young MSCs from *LMNB1^△Prx1^* mice at the age of 2 months showed an enhanced proportion of tubulated mitochondria, similar to senile *SLC7A11^fl/fl^* MSCs, and this phenotype was restored in MSCs from dCKO mice (Figure 6B). Electron microscopy confirmed the same pattern of mitochondrial morphological changes across all experimental groups (Figure 6C). Mitochondrial morphology is regulated by the dynamic process of fission and fusion, and an abnormally increased proportion of tubulated mitochondria indicates the disruption of fission, which can subsequently impede mitophagy and mitochondrial function [20]. Using the MT‐mKeima‐Red‐Mito lentivirus (mt‐Keima) to track mitophagy, we found that senile *SLC7A11^fl/fl^* MSCs and young *LMNB1^△Prx1^* MSCs exhibited weaker mitophagy, which was recovered in *SLC7A11^△Prx1^* MSCs and dCKO MSCs, respectively (Figure 6D). Consistently, impaired mitochondrial function, including enhanced intracellular ROS measured by CellROX, decreased mitochondrial membrane potential measured by TMRE staining and reduced oxidative phosphorylation (OXPHOS) measured by ATP and OCR assays, was shown in senile *SLC7A11 ^fl /fl^* MSCs and young *LMNB1^△Prx1^* MSCs. Mitochondrial dysfunction was also reversed by knocking out SLC7A11 in *SLC7A11^△Prx1^* MSCs and dCKO MSCs (Figure 6E–H). In addition, MASM7, a mitophagy inhibitor that disrupts mitochondrial fission, abolished the rescue effect of SLC7A11 knockout on the impaired osteogenesis of senile *SLC7A11 ^fl /fl^* MSCs and young *LMNB1^△Prx1^* MSCs (Figure 6I–L and Extended Data Figure 6). These findings reveal that fission disruption‐induced abnormalities in mitochondrial morphology and function are involved in the LMNB1‐SLC7A11‐mediated impairment of MSC osteogenesis.

**FIGURE 6:**
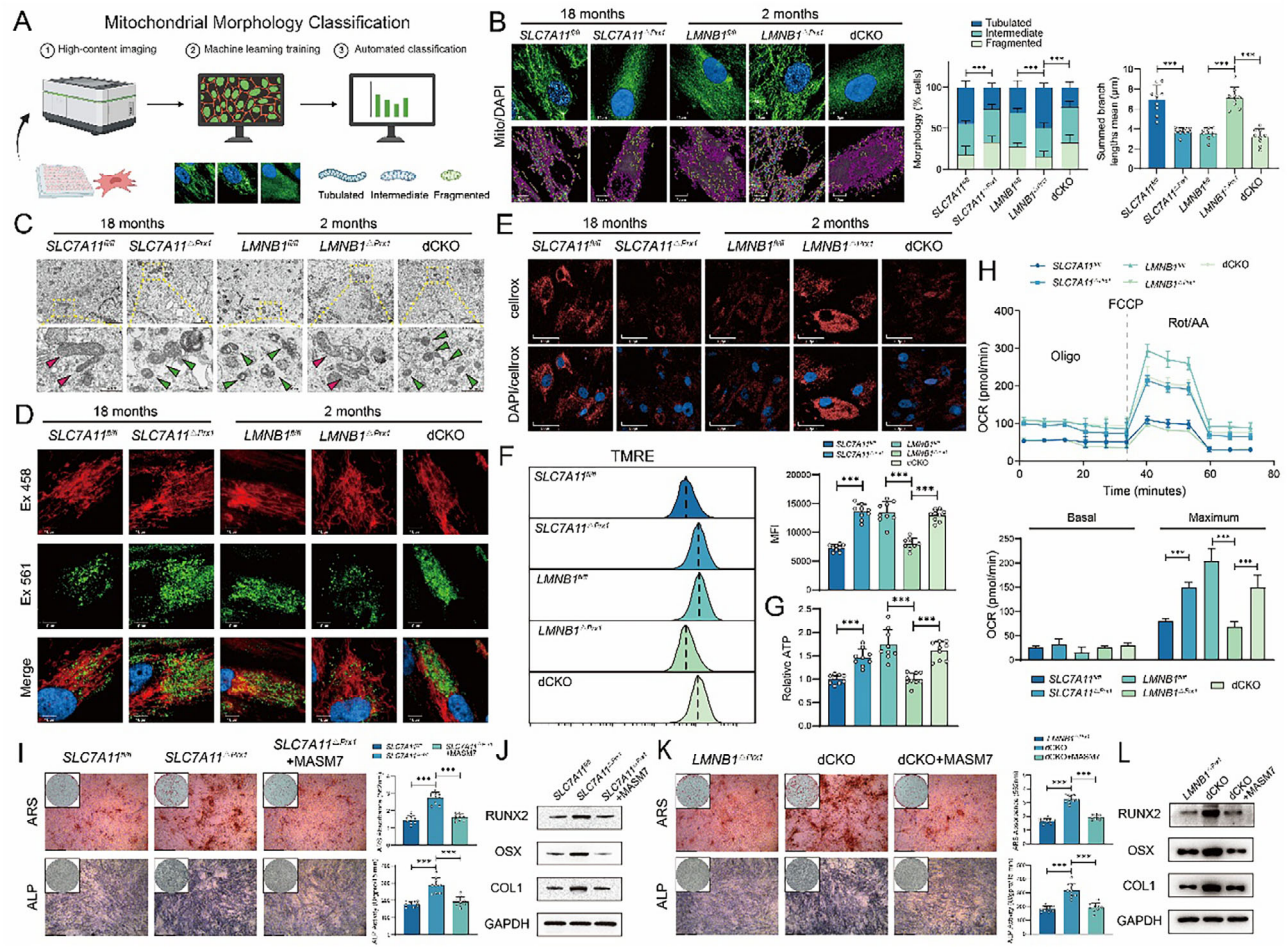
Fission disruption‐induced mitochondrial dysfunction is involved in the LMNB1‐SLC7A11‐mediated impairment of MSC osteogenesis. (A) Schematic diagram of the mitochondrial morphology classification procedure performed by a high‐content fluorescence imaging system. (B) High‐content fluorescence imaging of MitoTracker Green and mitochondrial network analysis of *SLC7A11 ^fl /fl^* or *SLC7A11^△Prx1^* MSCs extracted from 18‐month mice, *LMNB1^△Prx1^*, *LMNB1^fl/fl^* or dCKO MSCs extracted from 2‐month mice, and classification of mitochondrial morphology and summed branch lengths mean of mitochondria. (C) Representative images of mitochondria transmission electron microscopy in *SLC7A11 ^fl /fl^* or *SLC7A11^△Prx1^* MSCs extracted from 18‐month‐old mice and *LMNB1^△Prx1^*, *LMNB1^fl/fl^* or dCKO MSCs extracted from 2‐month‐old mice. (D) Representative images of mt‐Keima in *SLC7A11 ^fl /fl^* or *SLC7A11^△Prx1^* MSCs extracted from 18‐month‐old mice and *LMNB1^△Prx1^*, *LMNB1^fl/fl^* or dCKO MSCs extracted from 2‐month‐old mice. (E) Representative images of CellRox in *SLC7A11 ^fl /fl^* or *SLC7A11^△Prx1^* MSCs extracted from 18‐month‐old mice and *LMNB1^△Prx1^*, *LMNB1^fl/fl^* or dCKO MSCs extracted from 2‐month‐old mice. (F) TMRE flow cytometry analysis of *SLC7A11 ^fl /fl^* or *SLC7A11^△Prx1^* MSCs extracted from 18‐month‐old mice, *LMNB1^△Prx1^*, *LMNB1^fl/fl^* or dCKO MSCs extracted from 2‐month‐old mice, and quantitative analysis of TMRE MFI. (G) ATP measurement of *SLC7A11 ^fl /fl^* or *SLC7A11^△Prx1^* MSCs extracted from 18‐month‐old mice and *LMNB1^△Prx1^*, *LMNB1^fl/fl^* or dCKO MSCs extracted from 2‐month‐old mice. (H) OCR quantitative analysis of *SLC7A11 ^fl /fl^* or *SLC7A11^△Prx1^* MSCs extracted from 18‐month‐old mice and *LMNB1^△Prx1^*, *LMNB1^fl/fl^* or dCKO MSCs extracted from 2‐month‐old mice (n = 3). (I) ARS and ALP staining of *SLC7A11 ^fl/fl^* MSCs, *SLC7A11^△Prx1^* MSCs or *SLC7A11^△Prx1^* MSCs+MASM7 (1 µM) and quantitative analysis of ARS intensity and ALP activity. (J) Western blot analysis of RUNX2, OSX and COL I expression in *SLC7A11 ^fl /fl^* MSCs, *SLC7A11^△Prx1^* MSCs or *SLC7A11^△Prx1^* MSCs+MASM7. (K) ARS and ALP staining of *LMNB1^△Prx1^* MSCs, dCKO MSCs or dCKO MSCs+MASM7, and quantitative analysis of ARS intensity and ALP activity. (L) Western blot analysis of RUNX2, OSX and COL I expression in *LMNB1^△Prx1^* MSCs, dCKO MSCs or dCKO MSCs+MASM7. The data are presented as the means ± standard deviations (SD), n = 9 (unless specifically stated), ^*^ *p* < 0.05, ^**^ *p* < 0.01, ^***^ *p* < 0.005.

To further evaluate the role of disulfidptosis‐induced disulfide bond formation in actin in fission disruption‐induced mitochondrial dysfunction, we treated *LMNB1^△Prx1^* MSCs with TCEP, an inhibitor of disulfide bonds [16]. The results showed that TCEP treatment decreased the proportion of tubulated mitochondria (Figure 7A, B). In addition, mt‐Keima enhanced mitophagy in *LMNB1^△Prx1^* MSCs following TCEP treatment (Figure 7C). Enhanced intracellular ROS levels and decreased mitochondrial membrane potential and OXPHOS were restored after TCEP treatment in *LMNB1^△Prx1^* MSCs (Figure 7D–G). Similar results were confirmed in SOP‐MSCs, which exhibited a larger proportion of tubulated mitochondria, decreased mitophagy, enhanced intracellular ROS and impaired mitochondrial membrane potential and OXPHOS compared to HD‐MSCs. Notably, the dysfunction of mitochondrial energy metabolism in SOP‐MSCs was rectified by TCEP treatment, indicating that disulfide bond formation in actin resulted in mitochondrial fission disruption and the subsequent dysfunction of SOP‐MSCs (Extended Data Figure 7).

**FIGURE 7:**
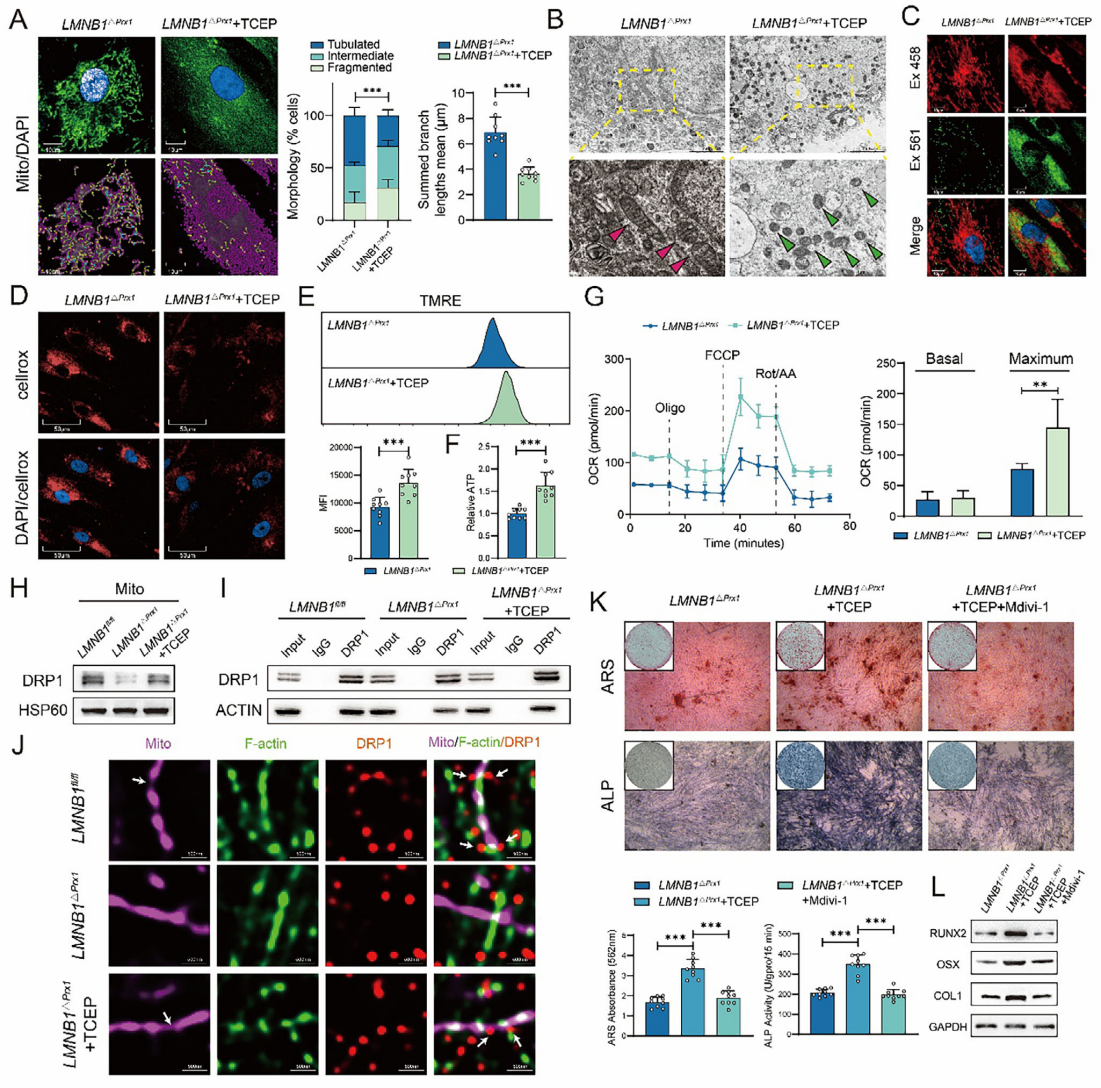
Disulfidptosis‐induced disulfide bond formation in actin disrupts DRP1‐mediated mitochondrial fission. (A) High‐content fluorescence imaging of MitoTracker Green and mitochondrial network analysis of *LMNB1^△Prx1^* MSCs or *LMNB1^△Prx1^* MSCs+TCEP (1 mM). and classification of mitochondrial morphology and summed branch lengths mean of mitochondria. (B) Representative images of mitochondrial transmission electron microscopy in *LMNB1^△Prx1^* MSCs or *LMNB1^△Prx1^* MSCs+TCEP. (C) Representative images of mt‐Keima in *LMNB1^△Prx1^* MSCs or *LMNB1^△Prx1^* MSCs+TCEP. (D) Representative images of CellRox in *LMNB1^△Prx1^* MSCs or *LMNB1^△Prx1^* MSCs+TCEP. (E) TMRE flow cytometry analysis of *LMNB1^△Prx1^* MSCs or *LMNB1^△Prx1^* MSCs+TCEP and quantitative analysis of TMRE MFI. (F) ATP measurement of *LMNB1^△Prx1^* MSCs or *LMNB1^△Prx1^* MSCs+TCEP. (G) OCR quantitative analysis of *LMNB1^△Prx1^* MSCs or *LMNB1^△Prx1^* MSCs+TCEP (n = 3). (H) Western blot analysis showing DRP1 expression in the mitochondria of *LMNB1^△Prx1^* MSCs or *LMNB1^△Prx1^* MSCs+TCEP. (I) Co‐IP analysis showing the binding of DRP1 and actin in *LMNB1^fl/fl^* MSCs, *LMNB1^△Prx1^* MSCs or *LMNB1^△Prx1^* MSCs+TCEP. (J) Representative images of MitoTracker DeepRed, FITC phalloidin and DRP1 immunofluorescence showing the colocalization of mitochondria, actin and DRP1 in *LMNB1^fl/fl^* MSCs, *LMNB1^△Prx1^* MSCs or *LMNB1^△Prx1^* MSCs+TCEP. (K) ARS and ALP staining of *LMNB1^△Prx1^* MSCs, *LMNB1^△Prx1^* MSCs+TCEP or *LMNB1^△Prx1^* MSCs+TCEP+Mdivi‐1 (10 µM) and quantitative analysis of ARS intensity and ALP activity. (L) Western blot analysis of RUNX2, OSX and COL I expression in *LMNB1^△Prx1^* MSCs, *LMNB1^△Prx1^* MSCs+TCEP or *LMNB1^△Prx1^* MSCs+TCEP+Mdivi‐1. The data are presented as the means ± standard deviations (SD), n = 9 (unless specifically stated), ^*^ *p* < 0.05, ^**^ *p* < 0.01, ^***^ *p* < 0.005.

Actin initiates mitochondrial fission by binding DRP1 to mitochondria for their restriction and separation [37]. Because no differences in the expression of mitochondrial fission and fusion markers, both at the mRNA and protein levels, were shown between SOP‐MSCs and HD‐MSCs or *LMNB1^△Prx1^* and *LMNB1^fl/fl^* MSCs (Extended Data Figure 8A–C), we hypothesize that disulfidptosis‐induced disulfide bond formation in actin and cytoskeletal collapse may impede the process of actin binding DRP1 to mitochondria. Mitochondria isolated from *LMNB1^△Prx1^* MSCs had significantly reduced protein levels of DRP1 compared with those from *LMNB1^fl/fl^* MSCs, and inhibiting the disulfide bond of actin using TCEP restored the mitochondrial DRP1 levels in *LMNB1^△Prx1^* MSCs, while TCEP treatment did not affect the mitochondrial fission and fusion marker expression of *LMNB1^△Prx1^* MSCs (Figure 7H, Extended Data Figure 8D, E). Co‐IP analysis demonstrated weakened binding between DRP1 and actin in *LMNB1^△Prx1^* MSCs compared to *LMNB1^fl/fl^* MSCs, which was restored upon TCEP treatment (Figure 7I). Super‐resolution fluorescence imaging confirmed the weakened binding among DRP1, actin and mitochondria in *LMNB1^△Prx1^* MSCs, and this phenomenon was eliminated with TCEP treatment (Figure 7J). Moreover, TCEP treatment restored the osteogenic differentiation of *LMNB1^△Prx1^* MSCs, while the DRP1 inhibitor Mdivi‐1 reversed this effect of TCEP (Figure 7K, L, Extended Data Figure 8F). Consistent results were shown in SOP‐MSCs (Extended Data Figure 8G–K). These results collectively demonstrated that disulfidptosis‐induced disulfide bond formation in actin disrupts DRP1‐mediated mitochondrial fission, therefore causing mitochondrial dysfunction to suppress SOP‐MSC osteogenesis.

**FIGURE 8:**
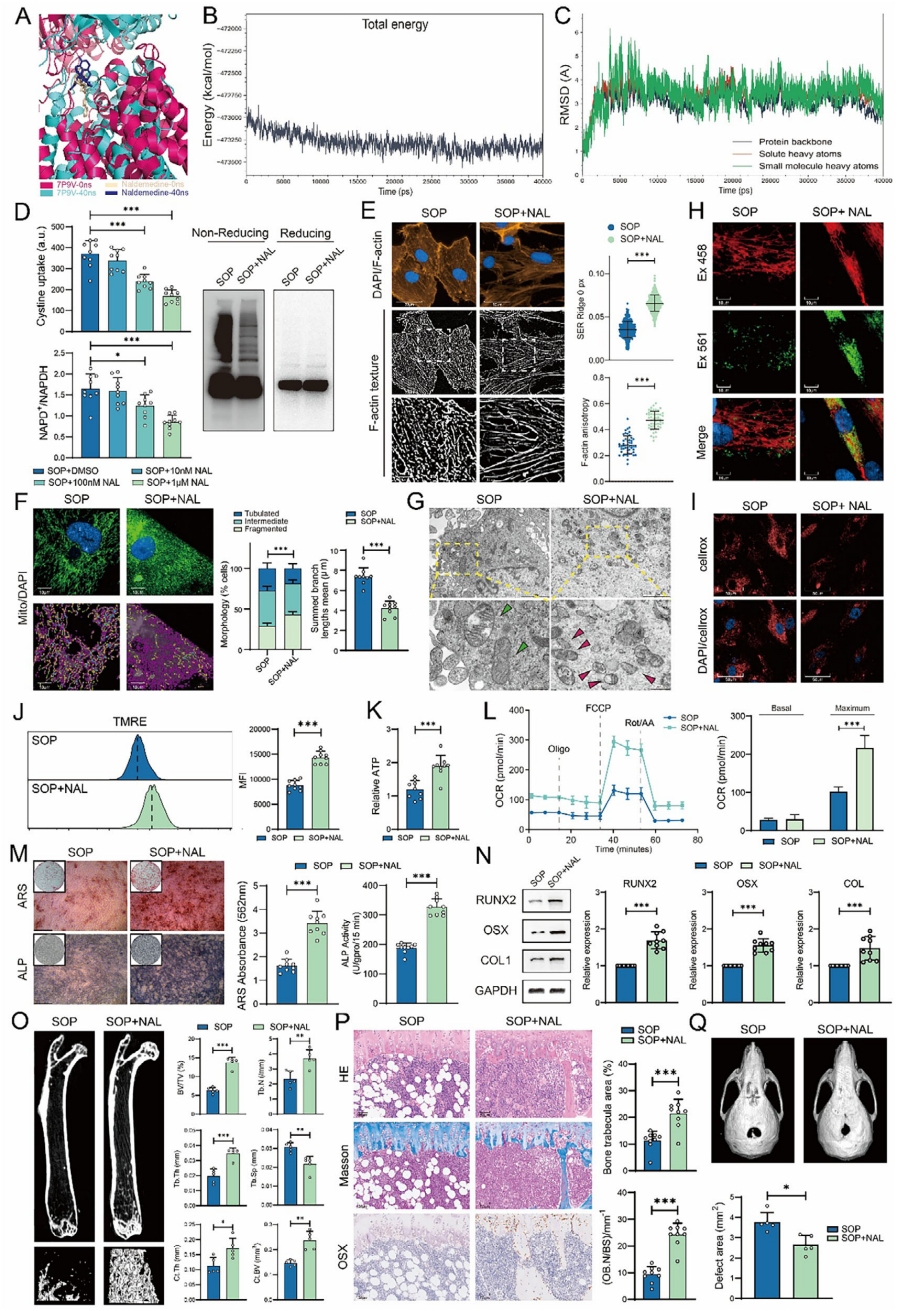
Naldemedine restores osteogenesis in SOP by targeting SLC7A11‐dependent disulfidptosis. (A) Molecular dynamics simulations of SLC7A11 and NAL. (B) Binding energy of the SLC7A11‐NAL complexes over time. (C) RMSD values of the SLC7A11‐NAL complexes over time. (D) Cystine uptake, NADP^+^/NADPH ratios and non‐reducing and reducing western blot analysis of actin in SOP‐MSCs+DMSO and SOP‐MSCs+NAL. (E) Representative TRITC‐Phalloidin immunofluorescence images captured by a high‐content analysis system showing the actin network texture of SOP‐MSCs+DMSO and SOP‐MSCs+NAL and actin morphology quantitative analysis of actin SER Ridge (n = 423–485 cells) and anisotropy (n = 50 cells). (F) High‐content fluorescence imaging of MitoTracker Green and mitochondrial network analysis of SOP‐MSCs+DMSO and SOP‐MSCs+NAL and classification of mitochondrial morphology and summed branch length mean of mitochondria. (G) Representative images of mitochondria transmission electron microscopy in SOP‐MSCs+DMSO and SOP‐MSCs+NAL. (H) Representative images of mt‐Keima in SOP‐MSCs+DMSO and SOP‐MSCs+NAL. (I) Representative images of CellRox in SOP‐MSCs+DMSO and SOP‐MSCs+NAL. (J) TMRE flow cytometry analysis of SOP‐MSCs+DMSO and SOP‐MSCs+NAL and quantitative analysis of TMRE MFI. (K) ATP measurement of SOP‐MSCs+DMSO and SOP‐MSCs+NAL. (L) OCR quantitative analysis of SOP‐MSCs+DMSO and SOP‐MSCs+NAL (n = 3). (M) ARS and ALP staining of SOP‐MSCs+DMSO and SOP‐MSCs+NAL and quantitative analysis of ARS intensity and ALP activity. (N) Western blot analysis of RUNX2, OSX and COL I expression in SOP‐MSCs+DMSO and SOP‐MSCs+NAL and relative quantitative analysis of RUNX2, OSX and COL I expression. (O) Representative micro‐CT images of femoral trabecular bone from SOP mice or SOP mice treated with NAL, with 3D reconstruction, and quantitative analysis of trabecular and cortical bone parameters in the femurs (n = 5 mice per group). (P) Histological sections of tibias from SOP mice or SOP mice treated with NAL stained with H&E, Masson's trichrome, and OSX immunohistochemistry and quantitative analysis of Masson‐positive area and OB.N/BS (n = 5 mice per group). (Q) Representative micro‐CT images showing calvarial defect healing in SOP mice or SOP mice treated with NAL and quantitative analysis of the calvarial defect areas (n = 5 mice per group). The data are presented as the means ± standard deviations (SD), n = 9 (unless specifically stated), ^*^ *p* < 0.05, ^**^ *p* < 0.01, ^***^ *p* < 0.005.

### 3.6 Naldemedine Serves as a Novel Anabolic Therapy for SOP by Targeting SLC7A11‐Mediated Disulfidptosis

The aforementioned experiments demonstrated that targeting SLC7A11‐dependent disulfidptosis in SOP‐MSCs restores their osteogenic capacity and exerts a significant therapeutic effect against SOP. To enhance the potential for clinical application and translational development, we performed structure‐based virtual screening of FDA‐approved drugs to identify specific inhibitors of SLC7A11 in clinical use. Three distinct protein structures of SLC7A11 (PDB: 7epz, 7p9u, 7p9v) were retrieved from the RCSB Protein Data Bank and used for molecular docking simulations against an FDA‐compound library. Using a binding energy threshold of < –10 kcal/mol, we identified three candidate drugs that could bind to all three SLC7A11 conformations, including glecaprevir (GLE), naldemedine (NAL), and dihydroergotamine (DIH) (Extended Data Figure 9A–C). Preliminary functional screening revealed that only NAL effectively intercepted disulfidptosis in SOP‐MSCs (Extended Data Figure 9D).

Molecular dynamics simulations using the cryo‐EM–resolved SLC7A11 structure (PDB: 7p9v) further confirmed the stable binding of NAL to SLC7A11 over a 40‐ns trajectory, indicating strong affinity and dynamic stability (Figure 8A–C). NAL treatment resulted in dose‐dependent interception of disulfidptosis in SOP‐MSCs and then restored the disulfide bond level of actin and the organized architecture of the cytoskeleton (Figure 8D, E, Extended Data Figure 10A). In addition, NAL‐treated SOP‐MSCs exhibited a decreased proportion of tubulated mitochondria and recovered mitophagy levels, accompanied by reduced ROS levels and enhanced mitochondrial membrane potential and OXPHOS (Figure 8F–L). In vitro osteogenesis assays showed that the osteogenic differentiation capacity of SOP‐MSCs was significantly strengthened upon NAL treatment (Figure 8M, N). Moreover, daily oral administration of NAL at a dose of 1 mg/kg over 30 days resulted in a substantial improvement in the bone mass of SOP mice, with increased bone trabecular area and OSX^+^ osteoblasts (Figure 8O, P). NAL treatment also markedly accelerated the healing process in SOP mice with calvarial defects (Figure 8Q). The safety of this therapeutic dose for SOP mice was assessed by the counts of red blood cells (RBCs), white blood cells (WBCs) and platelets (PLTs), QT interval and left ventricular ejection fraction (LVEF), serum alanine transaminase (ALT), aspartate transaminase (AST), total bilirubin (TBil), creatinine (CREA), urea nitrogen (BUN), amylase (AMY) and blood glucose, which were not affected by NAL treatment (Extended Data Figure 10B–F). These results strongly suggest that NAL rescued mitochondrial dysfunction and impaired osteogenesis of SOP‐MSCs by targeting SLC7A11‐mediated disulfidptosis, which can serve as a promising anabolic therapy for SOP.

## 4 Discussion

As the primary source of osteoblasts, the osteogenic differentiation capacity of bone marrow‐derived MSCs plays a crucial role in maintaining skeletal homeostasis, and the age‐related decline in their osteogenic potential represents a balance shifting in favor of bone resorption in SOP [38]. Identifying the age‐related pathogenic factor and revealing its detailed mechanism in impaired osteogenesis of MSCs provide new perspectives for developing osteoblast‐stimulating anabolic agents for SOP. LMNB1, a protein belonging to the lamin family protein, is considered a marker of cellular aging [39]. Previous studies determined that age‐dependent downregulation of LMNB1 impeded the function of stem cells and then led to the development of age‐related diseases [11, 30]. In the present study, we performed RNA‐seq to show the downregulation of LMNB1, rather than other lamin family molecules, including lamin A, lamin B2 and lamin C, in senile MSCs at passage 12. In addition, at the protein level, we confirmed that the LMNB1 downregulation of MSCs was aging dependent. By confirming the association between LMNB1 SNPs and osteoporosis susceptibility, we showed that LMNB1 expression was decreased in SOP‐MSCs and was positively related to their reduced osteogenic differentiation capacity. Moreover, the *LMNB1^△Prx1^* mice prematurely showed osteoporosis manifestations and delayed bone repair potential. These results primarily reveal the leading role of age‐related LMNB1 deficiency in the impaired osteogenesis of SOP.

As the main component of the nuclear lamina, a structural scaffold lining the inner nuclear membrane, LMNB1 participates in regulating diverse cellular processes through anchoring chromatin to the nuclear lamina to form LADs [9, 40]. Enriched with heterochromatin‐associated histone modifications, including H3K9me2 and H3K9me3, LADs maintain a closed chromatin structure that keeps most embedded genes in a transcriptionally silent or low‐expression state [41]. Although LADs cover a part of the chromatin, they encompass numerous development‐ and differentiation‐related genes, suggesting that dynamic LAD reorganization plays important roles in cellular differentiation and function [42, 43]. As recently reported, LAD redistribution not only releases adipogenic genes but also sequesters downregulated or repressed genes irrelevant to the adipose lineage [44]. Similarly, highly orchestrated reorganization of certain LADs progressively releases differentiation‐related genes to enable the programmed differentiation from embryonic to astrocytes [45]. In aging cells, LAD detachment promoted the ectopic expression of developmentally restricted genes, therefore accelerating senescence and impeding cellular functions [10, 46]. As shown by TEM, we clearly presented reduced chromatin anchoring at the nuclear periphery in both SOP‐MSCs and *LMNB1^△Prx1^* MSCs, indicating that LMNB1 deficiency caused LAD detachment in MSCs. This characteristic certainly led to heterochromatin detachment and structural alteration that was frequently accompanied by increased chromatin accessibility, as previously reported [47]. Our further analysis revealed an overall reduction in heterochromatin‐associated histone modifications (H3K9me2 and H3K9me3) alongside elevated levels of enhancer markers (BRD4, POL II, and H3K27ac) in SOP‐MSCs, which are considered features of aging cells [48]. As a transcriptional unbraked mechanism, these widespread epigenetic alterations resulted in abnormally enhanced expression of a series of genes related to actin cytoskeleton collapse, a prerequisite for the impaired osteogenesis of SOP‐MSCs that we have demonstrated.

Although LADs are generally transcriptionally repressed regions, the transcriptional activity of their bound genes varies, possibly because of their specific genetic elements and chromatin structure [49]. Moreover, the transcriptional levels of the LAD‐bound genes are also different after LAD detachment [50], suggesting that an additional precise mechanism drives transcriptional regulation of those most important genes following LAD detachment. SEs are functional clusters comprising multiple enhancer elements that recruit high‐density transcriptional complexes to drive robust expression of targeted genes [51]. As SEs are used to establish a position with accessible chromatin architecture [52], we postulated that LAD detachment‐mediated heterochromatin decompaction creates favorable conditions for SE formation, which then precisely activates the transcription of key osteogenesis‐suppressing genes and ultimately impairs the osteogenic differentiation of SOP‐MSCs. The present results confirmed this hypothesis by showing increased chromatin accessibility and the emergence of novel SEs at the sites of LAD detachment in SOP‐MSCs. In addition, although the SE profile is altered under aging conditions and is involved in the regulation of MSC function [53, 54], the upstream mechanism promoting SE formation and the location of chromatin structure are unclear. Our investigation provided new experimental evidence for addressing this issue, which is conducive to precise intervention targeting SEs.

Through integrated multi‐omics analysis and functional experiments, we identified that SLC7A11 was the sole key molecule that was upregulated by LMNB1 deficiency‐related SEs and caused impaired MSC osteogenesis in SOP. As a cystine/glutamate antiporter that facilitates cellular cystine uptake, SLC7A11 upregulation led to disulfidptosis in various cancer cells under glucose deprivation and NADPH depletion [55, 56]. Utilizing MSCs from SOP patients and three mouse models (*LMNB1^△Prx1^*, *SLC7A11^△Prx1^* and dCKO), we demonstrated that disulfidptosis activation was the key mechanism by which SLC7A11 upregulation impaired the osteogenic differentiation potential of SOP‐MSCs and accelerated bone loss in vivo. The key distinction lies in the fact that SOP‐MSCs do not require extracellular glucose deprivation to trigger disulfidptosis in our culture system, whereas SLC7A11‐high tumor cells often undergo disulfidptosis depending on glucose deprivation and NADPH depletion. This can be explained by the significant impairment of glucose uptake in senescent cells. Senescent MSCs undergo metabolic reprogramming that intrinsically restricts glucose influx, effectively creating a state of intracellular glucose starvation even within a glucose‐abundant microenvironment [57, 58]. This restricted intracellular glucose availability indicates impaired overall glucose utilization and reductive capacity, which likely attenuates the pentose phosphate pathway and limits baseline NADPH production. As NADPH depletion has been verified in aging cells, including MSCs [59, 60], we propose that age‐related NADPH deficiency creates a permissive environment for disulfidptosis activation in SOP‐MSCs. Another issue that confuses us compared with cancer studies is that SLC7A11 is usually upregulated in cancer cells to protect against oxidative stress‐induced ferroptosis [61], serving as a bridge linking ferroptosis and disulfidptosis [62]. As ferroptosis was reported as a factor that promotes osteoporosis [63], the adaptive upregulation of SLC7A11 may exhibit a protective effect on osteoblasts, while MSCs from *SLC7A11^△Prx1^* mice at the age of 18 months showed reduced osteogenic potential. It is possible that SLC7A11 may have a double‐edged sword effect. In the early stage of bone loss, SLC7A11 expression in MSCs increases to impede ferroptosis, and with aging and disease progression, cumulative SLC7A11 upregulation results in NADPH depletion and ultimately causes disulfidptosis to impair the osteogenesis of MSCs. This hypothesis still needs investigation in future studies.

The primary effect of disulfidptosis is to induce cytoskeletal collapse by promoting disulfide bond formation in actin, thereby inhibiting cancer cell growth and migration [16]. Organized actin architecture is particularly crucial for osteogenic differentiation, and disrupted actin networks result in the decreased osteogenic potential of MSCs [64]. In our study, we showed that actin cytoskeleton collapse resulting from disulfidptosis‐mediated disulfide bond formation impaired the osteogenesis of SOP‐MSCs and *LMNB1^△Prx1^* MSCs, and blocking disulfidptosis or conditionally knocking out SLC7A11 effectively reversed this effect. In particular, distinct actin structures greatly affect mitochondrial biology, and one well‐studied role of actin is to regulate mitochondrial fission [65]. Using high‐content microscopy with machine learning, we determined that mitochondrial fission in SOP‐MSCs and *LMNB1^△Prx1^* MSCs was significantly disrupted and then led to damaged mitophagy and OXPHOS, which are critical factors in ensuring the osteogenic differentiation of MSCs [66]. Our data elucidate a sequential link from disulfidptosis to a disorganized actin cytoskeleton, mitochondrial dynamics and cellular function in age‐related diseases. Moreover, in terms of the mechanism, we demonstrated that disulfidptosis‐mediated disulfide bond formation of actin restricted their interaction with DRP1, a cytosolic protein that oligomerizes into ring‐like structures and drives mitochondrial fission by constricting the membrane [67], and subsequently intercepted mitochondrial fission. Although this mechanism has not been extensively reported, it may represent a common mechanism through which disulfidptosis compromises mitochondrial dynamics.

Recent studies have consistently shown that osteoanabolic therapies targeting osteoblasts exhibit greater and more rapid reductions in fracture risk than antiresorptive treatments, but the limited diversity and clinical adverse effects indicate that the development of innovative osteoanabolic therapies remains essential [68]. Using both *SLC7A11^△Prx1^* and dCKO mouse models, we demonstrated that specifically inhibiting SLC7A11 significantly and effectively restored age‐related bone mass in vivo, indicating that it is a novel therapeutic target for SOP. Utilizing molecular docking‐based virtual screening with three different structural models of SLC7A11, we further identified that naldemedine, a selective peripheral opioid receptor antagonist clinically used for the treatment of opioid‐induced constipation in cancer patients [69], could be a candidate for targeting SLC7A11. Through in vitro and in vivo experiments, we confirmed that naldemedine successfully antagonized SLC7A11‐mediated disulfidptosis and the subsequent negative effects, therefore restoring the osteogenesis of SOP‐MSCs and age‐related bone loss. Importantly, naldemedine offers a therapeutic safety window by moderately inhibiting SLC7A11, which effectively prevents disulfidptosis without depleting the baseline antioxidant defense necessary to avert ferroptosis. While our functional assays suggest off‐target modulation, establishing definitive direct target engagement between naldemedine and SLC7A11 requires further biophysical validation. Additionally, although the 1 mg/kg in vivo dose provides robust proof‐of‐concept efficacy in our mouse model, direct extrapolation of this preclinical dosage to human clinical regimens requires caution. Although its safety was preliminarily detected in a mouse model, the potential risk of triggering ferroptosis via excessive SLC7A11 inhibition underscores the critical need for careful dose‐titration. Therefore, the clinical efficacy and long‐term safety of this drug require confirmation through a substantial number of well‐designed clinical studies.

In summary, this study reveals the connection among age‐related LMNB1 deficiency, SLC7A11‐dependent disulfidptosis and mitochondrial fission disruption as well as their pathological role in the impaired osteogenesis of SOP, which may provide new insight into developing a promising bone anabolic therapeutic strategy.

Several limitations remain, such as the double‐edged sword effect of SLC7A11 in ferroptosis and disulfidptosis, the detailed mechanism by which the actin disulfide bond disrupts DRP1 binding and the clinical role of naldemedine in SOP treatment. Furthermore, while our current findings highlight an MSC‐specific vulnerability, fully mapping the potential occurrence of disulfidptosis across diverse immune cell subpopulations or within the osteocytic network remains unexplored. It should also be noted that the *Prx1‐Cre* used in our study targets the broader limb mesenchyme lineage rather than being strictly MSC‐specific. Therefore, utilizing more highly restricted MSC‐specific models is warranted to further refine these lineage‐specific mechanisms. These questions warrant further investigation in future studies.

## Author Contributions

W.Y. and Q.L. wrote the manuscript. W.Z. prepared the figures and graphical presentations. W.Y. performed bioinformatic analyses and designed experiments. W.Z., G.Z., J.C. and Y.Z. conducted cellular experiments and mechanistic investigations. Z.X. (Zipeng Xiao) and Z.C. managed mouse breeding. Z.L. and Y.L. (Yangfeng Lin) performed animal experiments. J.L., Z.S. and Z.X. (Zhongyu Xie) reviewed and edited the manuscript. Z.X. (Zhongyu Xie) supervised the entire research project.

## Conflicts of Interest

The authors declare no conflicts of interest.

## Supporting information




**Supporting File 1**: advs78203‐sup‐0001‐SuppMat.docx.

**Supporting File 2**: advs78203‐sup‐0002‐TableS1.xlsx.

**Supporting File 3**: advs78203‐sup‐0003‐TableS2.xlsx.

**Supporting File 4**: advs78203‐sup‐0004‐TableS3.xlsx.

## Data Availability

We generated RNA‐seq data for human SOP‐MSCs vs healthy donor MSCs (HD‐MSCs), ATAC‐seq and CUT&Tag data (LMNB1, H3K27ac, BRD4, POL II, H3K9me2, H3K9me3) for human SOP‐MSCs vs HD‐MSCs, and the raw data have been deposited in the Genome Sequence Archive (Genomics, Proteomics & Bioinformatics 2025) in the National Genomics Data Center (Nucleic Acids Res 2025), China National Center for Bioinformation/Beijing Institute of Genomics, Chinese Academy of Sciences (GSA‐Human: HRA014532), which are publicly accessible at https://ngdc.cncb.ac.cn/gsa‐human (Shared URL: https://ngdc.cncb.ac.cn/gsa‐human/s/aC23Aicb). LMNB1 SNP data were obtained from the National Human Genome Research Institute (NHGRI) GWAS catalog (EFO_0004512), RNA‐seq data for P12 vs P4 MSCs were obtained from GEO (GSE178514), RNA‐seq data for SOP vs HD MSCs were obtained from SRA (PRJNA763497), and ChIP‐seq data for osteogenic progenitor cells were obtained from ChIP‐Atlas (https://chip‐atlas.org/).
