# Supplementary material for: Age‐Related Lamin B1 Deficiency Activates SLC7A11‐Dependent Disulfidptosis to Impair Osteogenesis via Intercepting Mitochondrial Fission
Source: Adv Sci (Weinh). 2026 Oct 8:e78203. Online ahead of print. doi: 10.1002/advs.78203 (PMC13650031; doi:10.1002/advs.78203)
Supplement: Supplementary file 1 [file ADVS-9999-e78203-s001.docx]

**Supplementary Materials For**

**Age-related Lamin B1 Deficiency Activates SLC7A11-Dependent Disulfidptosis to Impair Osteogenesis via** **Intercepting Mitochondrial Fission**

Wenhui Yu^1, 3*^, Qibo Li^1*^, Weihao Zhang^1*^, Guan Zheng^1,3^, Junhua chen^1^,

Yipeng Zeng^1^, Zipeng Xiao^1^, Zibin Chen^1^, Ziqian Liu^1^, Yangfeng Lin^1^,

Jiajie Lin^1#^, Zepeng Su^1#^, Zhongyu Xie^1,2,3#^

**Extended Data Fig. 1**

(A) Construction strategy for *LMNB1^△Prx1^* mice.
(B) Genotyping of *LMNB1^△Prx1^* mice.
(C) Western blot analysis of LMNB1 expression in the heart, liver, kidney, lung, femur and calvaria from *LMNB1^fl/fl^* or *LMNB1^△Prx1^* mice and relative quantitative analysis of LMNB1 expression.
(D) Representative immunofluorescence images of bone tissues verifying LMNB1 conditional knockout in MSCs.
(E) Relative quantitative analysis of RUNX2, OSX and COL I expression in MSCs from *LMNB1^fl/fl^* or *LMNB1^△Prx1^* mice during osteogenic differentiation.
(F) Relative quantitative analysis of LMNB1 expression in MSCs extracted from mice aged 2, 4, 8, 12, and 18 months during osteogenic differentiation.
(G) Relative quantitative analysis of LMNB1 expression in MSCs at passages 3, 6, 9, 12 and 15 during osteogenic differentiation.
(H) Relative quantitative analysis of LMNB1 expression in HD-MSCs and SOP-MSCs during osteogenic differentiation.
(I) Linear regression analysis between LMNB1 expression levels and ARS staining intensity.
(J) Quantitative analysis of ARS and ALP staining of osteogenic differentiating HD-MSCs+sh-NC, HD-MSCs+sh-LMNB1, SOP-MSCs+ov-NC and SOP-MSCs+ov-LMNB1.
(K) Relative quantitative analysis of RUNX2, OSX, COL I and LMNB1 expression in HD-MSCs+sh-NC, HD-MSCs+sh-LMNB1, SOP-MSCs+ov-NC and SOP-MSCs+ov-LMNB1 during osteogenic differentiation.

The data are presented as the means ± standard deviations (SD), n = 9 (unless stated), *P < 0.05, **P < 0.01, ***P < 0.005.

**Extended Data Fig. 2**

(A) Actin morphology quantitative analysis of actin SER Ridge (n = 361-507 cells) and anisotropy (n = 50 cells) of HD-MSCs+sh-NC, HD-MSCs+sh-LMNB1, SOP-MSCs+ov-NC and SOP-MSCs+ov-LMNB1.
(B) Actin morphology quantitative analysis of actin SER Ridge (n = 336-613 cells) and anisotropy (n = 50 cells) of MSCs treated with control vector, sh-LMNB1 or sh-LMNB1+jasplakinolide.
(C) Relative quantitative analysis of RUNX2, OSX and COL I expression in MSCs treated with sh-NC, sh-LMNB1 or sh-LMNB1+jasplakinolide during osteogenic differentiation.
(D) Representative TRITC-Phalloidin immunofluorescence images captured by a high-content analysis system showing the actin network texture of *LMNB1^fl/fl^* MSCs, *LMNB1^△Prx1^* MSCs+ov-NC, *LMNB1^△Prx1^* MSCs+ov-LMNB1 or *LMNB1^△Prx1^* MSCs+jasplakinolide, and actin morphology quantitative analysis of actin SER Ridge (n = 493-806 cells) and anisotropy (n = 50 cells).
(E) ARS and ALP staining of osteogenic differentiating *LMNB1^fl/fl^* MSCs, *LMNB1^△Prx1^* MSCs+ov-NC, *LMNB1^△Prx1^* MSCs+ov-LMNB1 or *LMNB1^△Prx1^* MSCs+jasplakinolide and quantitative analysis of ARS intensity and ALP activity.
(F) Western blot analysis of RUNX2, OSX, and COL I expression in osteogenic differentiating *LMNB1^fl/fl^* MSCs, *LMNB1^△Prx1^* MSCs+ov-NC, *LMNB1^△Prx1^* MSCs+ov-LMNB1 or *LMNB1^△Prx1^* MSCs+jasplakinolide and relative quantitative analysis of RUNX2, OSX and COL I expression.

The data are presented as the means ± standard deviations (SD), n = 9 (unless stated), *P < 0.05, **P < 0.01, ***P < 0.005.

**Extended Data Fig. 3**

(A) Relative quantitative analysis of H3K27ac, BRD4, POL II, H3K9me2 and H3K9me3 expression in HD-MSCs and SOP-MSCs and MSCs from *LMNB1^fl/fl^* or *LMNB1^△Prx1^* mice during osteogenic differentiation.
(B) Identification of SEs in HD-MSCs and SOP-MSCs using H3K27ac and BRD4 CUT&Tag via the ROSE algorithm
(C) Distribution of ATAC-seq and CUT&Tag peaks at different genomic elements.
(D) Positional relation with promoters of ATAC-seq and CUT&Tag peaks.

The data are presented as the means ± standard deviations (SD), n = 9 (unless specifically stated), *P < 0.05, **P < 0.01, ***P < 0.005.

**Extended Data Fig. 4**

(A) Relative quantitative analysis of LMNB1 and SLC7A11 expression in HD-MSCs+sh-NC, HD-MSCs+sh-LMNB1, SOP-MSCs+ov-NC and SOP-MSCs+ov-LMNB1 during osteogenic differentiation.
(B) ChIP-seq data of enhancer markers (H3K27ac and H3K4me1) and ATAC-seq data of multiple murine osteogenic progenitor cells at SLC7A11 loci (endosteal mesenchymal stromal cells, IDG-SW3, skeletal stem cells, bone cartilage and stromal progenitor cells, MC3T3-E1, osteogenic progenitors and skeletal progenitors).
(C) Schematic diagram showing the working mechanism by which the enCRISPRi system inhibits enhancer formation.
(D) Relative quantitative analysis of LMNB1 and SLC7A11 expression in *LMNB1^fl/fl^* MSCs*, LMNB1^fl/fl^* MSCs+enCRISPRi-1 and *LMNB1^fl/fl^* MSCs+enCRISPRi-2, *LMNB1^△Prx1^* MSCs, *LMNB1^△Prx1^* MSCs+enCRISPRi-1 and *LMNB1^△Prx1^* MSCs+enCRISPRi-2 during osteogenic differentiation.
(E) Relative quantitative analysis of RUNX2, OSX and COL I expression in *LMNB1^△Prx1^* MSCs, *LMNB1^△Prx1^* MSCs+enCRISPRi-1 and *LMNB1^△Prx1^* MSCs+enCRISPRi-2 during osteogenic differentiation.
(F) Constructing strategy for *SLC7A11^△Prx1^* mice.
(G) Genotyping of *SLC7A11^△Prx1^* mice.
(H) Western blot analysis of SLC7A11 expression in the heart, liver, kidney, lung, femur and calvaria from *SLC7A11^fl/fl^* and *SLC7A11^△Prx1^*mice, and relative quantitative analysis of SLC7A11 expression.
(I) Representative immunofluorescence images of bone tissues verifying SLC7A11 conditional knockout in MSCs.
(J) Relative quantitative analysis of RUNX2, OSX and COL I expression in *SLC7A11^fl/fl^* MSCs, *SLC7A11^△Prx1^* MSCs or *SLC7A11^△Prx1^* MSCs+CytoD during osteogenic differentiation.

The data are presented as the means ± standard deviations (SD), n = 9 (unless specifically stated), *P < 0.05, **P < 0.01, ***P < 0.005.

**Extended Data Fig. 5**

(A) Flow cytometry analysis of PI staining showing the cell death rates of SOP-MSCs treated with 100 μM, 200 μM, 400 μM or 800 μM cystine.
(B) Relative quantitative analysis of RUNX2, OSX and COL I expression in SOP-MSCs treated with 100 μM, 200 μM, 400 μM or 800 μM cystine during osteogenic differentiation.
(C) Flow cytometry analysis of PI staining showing the cell death rates of HD-MSCs, SOP-MSCs or SOP-MSCs+NAC.
(D) Flow cytometry analysis of PI staining showing the cell death rates of *LMNB1^fl/fl^* MSCs and *LMNB1^△Prx1^* MSCs.
(E) Flow cytometry analysis of PI staining showing the cell death rates of MSCs from 18-month *SLC7A11^fl/fl^* or *SLC7A11^△Prx1^* mice.
(F) Flow cytometry analysis of PI staining showing the cell death rates of *LMNB1^fl/fl^*, *LMNB1^△Prx1^* or dCKO MSCs cultured with 200 μM cystine and *LMNB1^△Prx1^* or dCKO MSCs cultured with 800 μM cystine.
(G) Relative quantitative analysis of RUNX2, OSX, COL I, SLC7A11 and LMNB1 expression in *LMNB1^fl/fl^*, *LMNB1^△Prx1^* or dCKO MSCs cultured with 200 μM cystine and *LMNB1^△Prx1^* or dCKO MSCs cultured with 800 μM cystine.

The data are presented as the means ± standard deviations (SD), n = 9 (unless specifically stated), *P < 0.05, **P < 0.01, ***P < 0.005.

**Extended Data Fig. 6**

(A) Relative quantitative analysis of RUNX2, OSX and COL I expression in *SLC7A11^f1/fl^* MSCs, *SLC7A11^△Prx1^* MSCs or *SLC7A11^△Prx1^* MSCs+MASM7.
(B) Relative quantitative analysis of RUNX2, OSX and COL I expression in *LMNB1^△Prx1^* MSCs, dCKO MSCs and dCKO MSCs+MASM7.

The data are presented as the means ± standard deviations (SD), n = 9 (unless specifically stated), *P < 0.05, **P < 0.01, ***P < 0.005.

**Extended Data Fig. 7**

(A) High-content fluorescence imaging of MitoTracker Green and mitochondrial network analysis of HD-MSCs, SOP-MSCs and SOP-MSCs+TCEP and classification of mitochondrial morphology and summed branch lengths mean of mitochondria.
(B) Representative images of mitochondria transmission electron microscopy in HD-MSCs, SOP-MSCs and SOP-MSCs+TCEP.
(C) Representative images of mt-Keima in HD-MSCs, SOP-MSCs and SOP-MSCs+TCEP
(D) Representative images of CellRox in HD-MSCs, SOP-MSCs and SOP-MSCs+TCEP
(E) TMRE flow cytometry analysis of HD-MSCs, SOP-MSCs and SOP-MSCs+TCEP and quantitative analysis of TMRE MFI.
(F) ATP measurement of HD-MSCs, SOP-MSCs and SOP-MSCs+TCEP.
(G) OCR quantitative analysis of HD-MSCs, SOP-MSCs and SOP-MSCs+TCEP (n=3).

The data are presented as the means ± standard deviations (SD), n = 9 (unless specifically stated), *P < 0.05, **P < 0.01, ***P < 0.005.

**Extended Data Fig. 8**

(A) Volcano plot of RNA-seq data comparing SOP-MSCs and HD-MSCs during osteogenic differentiation showing the expression of mitochondrial fission- and fusion-associated genes.
(B) Western blot analysis of DRP1, FIS, MFN1 and MFN2 expression in HD-MSCs and SOP-MSCs and quantitative analysis of DRP1, FIS, MFN1 and MFN2 expression.
(C) Western blot analysis of DRP1, FIS, MFN1 and MFN2 expression in *LMNB1^fl/fl^* MSCs or *LMNB1^△Prx1^* MSCs and quantitative analysis of DRP1, FIS, MFN1 and MFN2 expression.
(D) Quantitative analysis of DRP1 expression in the mitochondria of *LMNB1^fl/fl^* MSCs or *LMNB1^△Prx1^* MSCs and *LMNB1^△Prx1^* MSCs+TCEP.
(E) Western blot analysis of DRP1, FIS, MFN1 and MFN2 expression in *LMNB1^△Prx1^* MSCs or *LMNB1^△Prx1^* MSCs+TCEP and quantitative analysis of DRP1, FIS, MFN1 and MFN2 expression.
(F) Quantitative analysis of RUNX2, OSX and COL I expression in *LMNB1^△Prx1^* MSCs or *LMNB1^△Prx1^* MSCs+TCEP.
(G) Western blot analysis showing DRP1 expression in the mitochondria of HD-MSCs, SOP-MSCs and SOP-MSCs+TCEP and quantitative analysis of DRP1 expression.
(H) Co-IP analysis showing the binding of DRP1 and actin in HD-MSCs, SOP-MSCs or SOP-MSCs+TCEP.
(I) Representative images of Mito-Tracker DeepRed, FITC phalloidin and DRP1 immunofluorescence showing the colocalization of mitochondria, actin and DRP1 in HD-MSCs, SOP-MSCs or SOP-MSCs+TCEP.
(J) ARS and ALP staining of SOP-MSCs, SOP-MSCs+TCEP or SOP-MSCs+TCEP+Mdivi-1 and quantitative analysis of ARS intensity and ALP activity.
(K) Western blot analysis of RUNX2, OSX and COL I expression in SOP-MSCs, SOP-MSCs+TCEP or SOP-MSCs+TCEP+Mdivi-1 and relative quantitative analysis of RUNX2, OSX and COL I expression.

The data are presented as the means ± standard deviations (SD), n = 9 (unless specifically stated), *P < 0.05, **P < 0.01, ***P < 0.005.

**Extended Data Fig. 9**

(A) List of FDA-approved drugs targeting SLC7A11 (binding affinity < -10 kcal/mol)
(B) Venn diagram showing the intersection of FDA-approved drugs that target the three distinct structures of SLC7A11.
(C) Molecular docking analysis showing the binding of SLC7A11 with GLE, DIH, and NAL.
(D) Cystine uptake, NADP^+^/NADPH ratios and flow cytometry analysis of PI staining showing the cell death rates of HD-MSCs+DMSO, SOP-MSCs+DMSO, SOP-MSCs+GLE (10 nM), SOP-MSCs+DIH (10 μM) and SOP-MSCs+NAL (1 μM).

**Extended Data Fig. 10**

(A) Flow cytometry analysis of PI staining showing the cell death rates of SOP-MSCs+DMSO, SOP-MSCs+10 nM NAL, SOP-MSCs+100 nM NAL and SOP-MSCs+1 μM NAL.
(B) Complete blood count analysis of SOP mice and SOP mice treated with NAL. RBCs, WBCs, and PLTs were measured to evaluate potential hematological toxicity.
(C) Cardiac function assessment of SOP mice and SOP mice treated with NAL. Electrocardiography was used to measure the QT interval, and echocardiography was performed to determine the LVEF.
(D) Hepatic function evaluation of SOP mice and SOP mice treated with NAL. Serum levels of ALT, AST, and TBil were measured as biomarkers of liver injury.
(E) Renal function and metabolic parameter analysis of SOP mice and SOP mice treated with NAL. Serum levels of CREA and BUN were quantified to assess renal function.
(F) Pancreatic function and metabolism analysis of SOP mice and SOP mice treated with NAL. Serum AMY activity and blood glucose levels were measured to evaluate pancreatic function and metabolic status.

**Extended data table**

Table S1 Clinical features of control and senile osteoporosis subjects

| Clinical features | Group | | P value |
| --- | --- | --- | --- |
|  | Control | Osteoporosis |  |
| All subjects (n, %) | 17 (100) | 23 (100) |  |
| Gender |  |  | 0.6760 |
| Male | 10 | 12 |  |
| Female | 7 | 11 |  |
| Age (mean ± sd) | 28.22±4.33 | 78.32±6.02 | <0.0001 |
| T score | -0.44±0.12 | -3.12±0.41 | <0.0001 |
| BMI (kg/m2) | 20.11±4.03 | 19.34±3.72 | 0.5359 |

Table S2 Table S2 Construct and reagents

| Construct | Application | Sequence (5’-3’) |
| --- | --- | --- |
| Lenti-sh-LMNB1 | LMNB1 knockdown | CGCTTGAAGAACACTTCTGAA |
| Lenti-sh-SLC7A11 | SLC7A11 knockdown | AGATATGCATCGTCCTTTCAA |
| Lenti-ov-LMNB1 | LMNB1 overexpression | / |
| Lenti-ov-SLC7A11 | SLC7A11 overexpression | / |
| AAV9-Flex-shSLC7A11 | In vivo conditional knockout SLC7A11 in MSCs | / |
| MT-mKeima-Red-Mito | Evaluate mitophagy levels | / |
| dCas9-KRAB | Inhibit the activation of enhancer | / |
| U6-sgRNA-1(MS2)-EF1a-NLS-MCP-LSD1-P2A-puro | Guiding dCas9-KRAB to deactivate specific enhancer | ATCACCAGCAGCGGGTACCA |
| U6-sgRNA-2(MS2)-EF1a-NLS-MCP-LSD1-P2A-puro | Guiding dCas9-KRAB to deactivate specific enhancer | GTTTCTGCACACGAGAGCCG |

Table S3 SEs signals of SOP-MSCs (see attached excel file)

Table S4 LADs detachment regions of SOP-MSCs (see attached excel file)

Table S5 Genes in Venn analysis of Figure 3A (see attached excel file)
